# Effectiveness of Nonfunctionalized Graphene Oxide Nanolayers as Nanomedicine against Colon, Cervical, and Breast Cancer Cells

**DOI:** 10.3390/ijms24119141

**Published:** 2023-05-23

**Authors:** Mohammad Rafe Hatshan, Quaiser Saquib, Maqsood A. Siddiqui, Mohammad Faisal, Javed Ahmad, Abdulaziz A. Al-Khedhairy, Mohammed Rafi Shaik, Mujeeb Khan, Rizwan Wahab, Valeria De Matteis, Syed Farooq Adil

**Affiliations:** 1 Department of Chemistry, College of Sciences, King Saud University, P.O. Box 2455, Riyadh 11451, Saudi Arabia; mhatshan@ksu.edu.sa (M.R.H.); mrshaik@ksu.edu.sa (M.R.S.); kmujeeb@ksu.edu.sa (M.K.); sfadil@ksu.edu.sa (S.F.A.); 2 Chair for DNA Research, Zoology Department, College of Sciences, King Saud University, P.O. Box 2455, Riyadh 11451, Saudi Arabia; maqsoodahmads@gmail.com (M.A.S.); javedbiochem@gmail.com (J.A.); kedhairy@ksu.edu.sa (A.A.A.-K.); rizwannano@gmail.com (R.W.); 3 Botany and Microbiology Department, College of Sciences, King Saud University, P.O. Box 2455, Riyadh 11451, Saudi Arabia; faisalm15@gmail.com; 4 Department of Mathematics and Physics “Ennio De Giorgi”, University of Salento, Via Arnesano, 73100 Lecce, Italy; valeria.dematteis@unisalento.it

**Keywords:** graphene oxide, graphene oxide nanolayers, anticancer, nanoparticles, apoptosis, cell death

## Abstract

Recent studies in nanomedicine have intensively explored the prospective applications of surface-tailored graphene oxide (GO) as anticancer entity. However, the efficacy of nonfunctionalized graphene oxide nanolayers (GRO-NLs) as an anticancer agent is less explored. In this study, we report the synthesis of GRO-NLs and their in vitro anticancer potential in breast (MCF-7), colon (HT-29), and cervical (HeLa) cancer cells. GRO-NLs-treated HT-29, HeLa, and MCF-7 cells showed cytotoxicity in the MTT and NRU assays via defects in mitochondrial functions and lysosomal activity. HT-29, HeLa, and MCF-7 cells treated with GRO-NLs exhibited substantial elevations in ROS, disturbances of the mitochondrial membrane potential, an influx of Ca^2+^, and apoptosis. The qPCR quantification showed the upregulation of *caspase 3*, *caspase 9*, *bax*, and *SOD1* genes in GRO-NLs-treated cells. Western blotting showed the depletion of P21, P53, and CDC25C proteins in the above cancer cell lines after GRO-NLs treatment, indicating its function as a mutagen to induce mutation in the *P53* gene, thereby affecting P53 protein and downstream effectors P21 and CDC25C. In addition, there may be a mechanism other than P53 mutation that controls P53 dysfunction. We conclude that nonfunctionalized GRO-NLs exhibit prospective biomedical application as a putative anticancer entity against colon, cervical, and breast cancers.

## 1. Introduction

Cancers are one of the major public health problems that caused the deaths of 99,58,133 patients globally in 2020, as reported by the WHO and IARC. In the same year, 19,29,2,789 new cases of cancer were detected on a worldwide basis [1]. In an attempt to provide a summary of current scientific information on various types of cancer in the USA, the American Cancer Society reported expecting 19,58,310 new cases of cancer and 6,09,820 deaths in male and female patients during the year 2023. Among the types of cancer, breast cancer may account for 3,00,590 new cases and 43,700 deaths. For uterine cervical cancers, 13,960 new cases and 4,310 deaths are expected. On the other hand, 1,53,020 new cases and 52,550 deaths are estimated for colon and rectal cancers [2]. Among the cohort of all cancers, breast cancer is an anthology of prominent diseases in the mammary gland, predominantly due to the inheritance of mutated breast cancer genes 1 or 2 (*BRCA1* or *BRCA2*) from parents, although several non-genetic aspects do play a critical role in the onset of breast cancer. Broadly, breast cancer has been classified into carcinomas and sarcomas. The former has been detected in the majority of cases, while the latter has been rarely seen [3]. In 2020, a total 6,84,996 deaths were recorded owing to breast cancer, and in parallel, 22,61,419 new cases were reported [1]. Early detection of breast cancer and improved comprehensive treatment lead to a greater chance of patient survival. Hence, there is an enhancement in the counts of patient survival at the stage of post-therapeutic healing [4,5]. Notwithstanding this fact, the reoccurrence of breast cancer after the first or second round of treatments is frequent, which leads to the death of patients mainly due to aggressive tumor biology. It is also concerning that normal breast cancer cells acquire the properties of cancer stem cells, which are reckoned to enhance cellular stability against chemo- and radiotherapies. Such properties prompt tumor heterogeneity as well as increase the quantity of breast cancer stem cells, causing therapeutic failure and reoccurrence of cancer [6].

Cervical cancer pathogenesis initiates through a slow process of interruption with normal differentiation of cervical squamous epithelium, eventually triggering structural and physiological alterations [7]. Worldwide deaths owing to cervical cancer reached 3,41,831, while 6,04,127 new cases of cervical cancer were detected in 2020 [1]. Cervical cancer has been categorized as the fourth prominent factor for cancer in women and is associated with greater mortality among them [8,9]. Women from developed and developing nations are prone to cervical cancer, and 90% of deaths occur in middle- and low-income countries [10]. The human papilloma virus is a causative agent for cervical cancer. Several factors, including epigenetic and genetic changes induced by smoking, HIV infection, and increased parity, are some of the prominent factors triggering cervical cancer [11,12,13,14]. Early diagnosis of cervical cancer has led to its cure through different therapeutic interventions [15].

In the same context, global deaths from colorectal cancer reached 9,35,173 cases, together with 19,31,590 new cases in 2020. In the same year, colorectal cancer was found to be the third largest cause of mortality and the fourth among new cancer cases in males and females of all age groups [1]. Increased risk for developing colorectal cancer is associated with modifiable factors, *viz*., tobacco, alcohol consumption (low to moderate), diet, absence of physical activity, and obesity. On the other hand, high-risk factors under non-modifiable factors include a familial history of colorectal cancer, inflammatory bowel disease, Lynch syndrome, type 2 diabetes, and racial or ethnic background [16]. Colorectal cancer often grows very slowly and produces no symptoms, unless the colon tumor reaches a considerable size via multistep changes in histological, morphological, and genetic makeup over time [16,17]. Biomarkers of early cancer detection and treatment approaches, such as chemotherapy, immunotherapy, targeted therapy, hormone therapy, and radiotherapy, have demonstrated substantial success in curing or reducing the burden of disease in patients. The microenvironment of tumors and cancerous cells is highly complex and dynamic due to the precise variations in their extracellular and cellular matrix compositions. These factors are liable for the adjustment of matrix stiffness [18]. In addition, other hurdles faced by clinicians when combating the life-threatening risk of cancer are multidrug resistance, inadequate influx of drugs into tumors, and complexity of chemotherapy [19,20,21]. Such constraints have prompted researchers and scientists globally to work on the production of new entities or materials that can be applied in medical sciences for effective treatment of cancers and bypass the issue of chemoresistance. 

Nanoparticles have unique physicochemical properties and sizes, which give them an upper hand in biomedical implementations, including bone regeneration, bio-imaging, wound healing, and targeted drug delivery systems. The nano-range dimensions specifically allow the interaction of nanoparticles with biological macromolecules in different ways [22]. Cancer cell lines and surface-functionalized nanoparticles have shown effective interactions via better pharmacokinetic potential as well as fewer adverse effects when such nanoparticles are used to circumvent human cancers [23,24]. Graphene is a 2D allotrope of carbon exhibiting a 1.42 Å in distance between adjoining carbon atoms. Fundamentally, graphene is a monolayer (one-atom thick layer of sp^2^ hybridized carbon) in a tightly packed honeycomb crystal lattice that possesses astonishing properties [25]. Graphene oxide (GO) physical and chemical attributes make it a suitable choice among nanomaterials for effective application in biomedical settings [26]. Nanocomposites based on metal–organic frameworks and graphene-based components exhibit benefits in the preparation of gas adsorbents [27]. In the current time, materials containing graphene are a favorable choice for the production of dental implants [28,29], scaffolds for tissue and muscle regeneration [29,30], biosensors [31], and drug delivery vehicles for anticancer pharmaceuticals [32]. The complexation of GO and GO quantum dots with curcumin demonstrated anticancer effects against breast cancer cell lines [33], whereas a nanocomposite of silver and GO showed better cellular uptake and cytotoxicity in cancer cells [34]. Additionally, a GO-PEGylated folate nanosystem showed targeted drug delivery in HeLa cells [35]. Reduced GO functionalized by polyethylene glycol exhibited anti-cancer effects by killing lung cancer cells (A549) [36]. GO nanosheets loaded with the flavonoid hesperidin significantly reduced colon cancer polyps in experimental rats by inhibiting cyclooxygenase-2 (COX-2) and inducible nitric oxide synthase (iNOS) pathways [37]. GO loaded with pirfenidone demonstrated efficacy against subarachnoid hemorrhage. GO possesses a unique surface area, which induces hydrophobic interactions as well as π–π stacking with pirfenidone to provide substantial benefits [38]. Doxorubicin loaded on a GO derivative affected the cell cycle of human multiple myeloma cells, ultimately causing their death [39]. The above-mentioned attempts revealed that application of graphene could be achieved for drug delivery, especially for poorly water-soluble drugs, without affecting its efficiency [40].

In view of the above evidences, graphene and its derivatives appear to bring a new ray of hope for tumor therapy. Nonetheless, the application of nonfunctionalized graphene oxide nanolayers (GRO-NLs) as an anti-cancer entity is still in its infancy. Consequently, we report the synthesis of GRO-NLs and evaluate their anticancer efficacies by measuring their cytotoxicity, oxidative stress, mitochondrial dysfunction, Ca^2+^ influx, and apoptosis in MCF-7, HT-29, and HeLa cells.

## 2. Results

### 2.1. GRO-NLs Characterization

#### 2.1.1. X-ray Diffraction and Thermal Property

**Figure 1:**
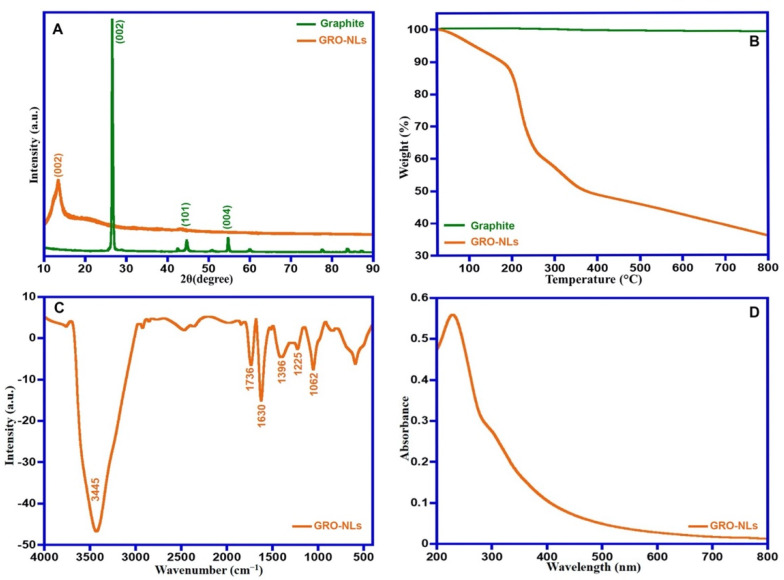
Characterization of nonfunctionalized GRO-NLs: (**A**) PXRD patterns of pristine graphite and GRO-NLs, (**B**) TGA thermograph of graphite and GRO-NLs, (**C**) FTIR spectrum of GRO-NLs, and (**D**) UV-visible absorbance spectrum of GRO-NLs.

The crystallographic structure of pure graphite and GRO-NLs was analyzed using powder X-ray diffraction (PXRD) (Figure 1A). The pristine graphite displays a sharp reflection centered at 26.6°, which is equivalent to the (0 0 2) lattice plane, along with an interlayer space of 3.42 Å. While the appearance of fingerprint broad reflection of the GRO-NLs was recorded around 11.6°, which is related to the (0 0 2) lattice plane. The thermal properties of the GRO-NLs and their precursor graphite were tested by utilizing TGA (Figure 1B). The synthesized materials were subjected to a range of heating temperatures (25 °C–800 °C) with a 10 °C/min heating rate in an N_2_ atmosphere. The graphite’s TGA curve displays the entire mass loss (1%). In contrast, the GRO-NLs were thermally uneven; the GRO-NLs began to evaporate the absorbed water as well as the volatile materials at 100 °C, followed by a main mass loss of 44% in the range of 205 °C to 365 °C. Ultimately, about 11% of the GRO-NLs’ weight vanished between 365 °C and 800 °C.

#### 2.1.2. FTIR, UV-Vis, Raman Spectra and HR-TEM of GRO-NLs

**Figure 2:**
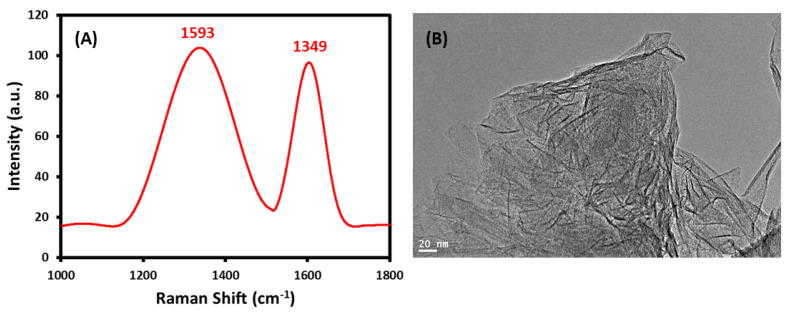
Characterization of nonfunctionalized GRO-NLs: (**A**) Raman spectrum of GRO-NLs showing two fingerprint bands, and (**B**) HR-TEM microscopic image of GRO-NLs.

The FTIR spectrum of the GRO-NLs shows a wide-ranging band at ~3445 cm^−1^ owing to OH group stretching vibration and the existence of oxygen-possessing groups (Figure 1C). The featured peaks assigned to –COOH groups are noticeable at 1736 cm^−1^, and C=C/C–C from the graphitic region at 1630 cm^−1^. The bands located at 1396 cm^−1^, 1225 cm^−1^, and 1062 cm^−1^ are attributed to C–OH, C–O–C, and C–O vibrations. In addition, the GRO-NLs UV-vis spectrum displays two characteristic absorption bands at approximately 230 nm and 301 nm (Figure 1D). Figure 2A describes the Raman pattern of the GRO-NLs. The Raman pattern of the GRO-NLs possesses two fingerprint bands positioned at 1593 cm^−1^ and 1349 cm^−1^, often symbolized as G- and D-bands. The morphology of the GRO-NLs was examined through HR-TEM microscopy, which displays extremely exfoliated nanolayers that resemble thin, transparent flakes (Figure 2B).

### 2.2. Cytotoxic Effects of GRO-NLs

**Figure 3:**
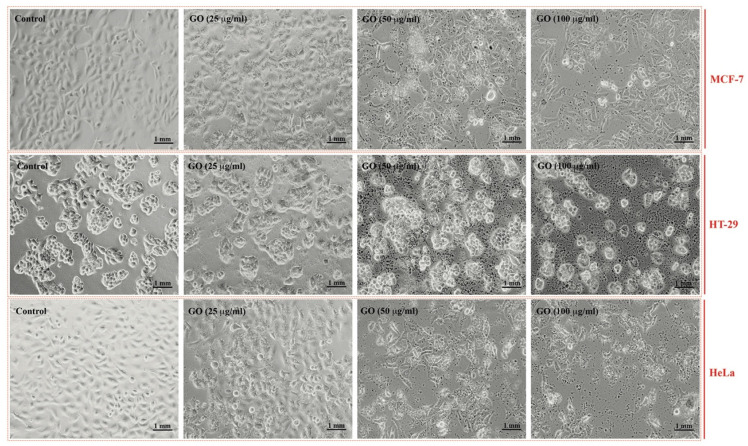
Effect of GRO-NLs on the survival of three cell lines. Representative light microscopic images showing morphological changes in the cancer cells after exposure to GRO-NLs for 24 h. The images were captured at 20× magnification.

The cell lines treated with GRO-NLs exhibited morphological changes. The changes were expressed in the form of gaps between adjacent cells, shrinkage, and cellular detachment post-24 h exposure (Figure 3). The MTT data showed cytotoxicity in all cell lines post-GRO-NLs treatment. Relative to 100% proliferation in the control, MCF-7 proliferation significantly decreased to 15.1%, 21.0%, and 32.7% after exposure to different concentrations of GRO-NLs (25, 50, and 100 μg/mL). At a similar concentration range of GRO-NLs, HT-29 and HeLa cells exhibited 22.8%, 48.0%, 57.1%, and 17.8%, 35.5%, and 44.4% decreased cell proliferation, respectively (Supplementary Figure S1). 

The cytotoxic effects of GRO-NLs via disturbances in lysosome function also showed a significant decline in the viability of the cell lines. MCF-7 proliferation significantly decreased to 12.9% after exposure to 100 μg/mL of GRO-NLs. Exposure of HT-29 cells to 25–100 μg/mL of GRO-NLs affected proliferation by declining cell survival to 33.7%, 41.4%, and 43.8%, compared to the 100% proliferation in the control. HeLa cells exposed to GRO-NLs (25–100 μg/mL) showed a 27.1%, 42.2%, and 512.4% decline in survival (Supplementary Figure S3). The GRO-NLs IC50 in MCF-7, HT29, and HeLa cells analyzed via the log inhibitor versus normalized response variable slope using the MTT and NRU data are shown in Supplementary Figures S2 and S4.

### 2.3. Apoptosis Quantification

**Figure 4:**
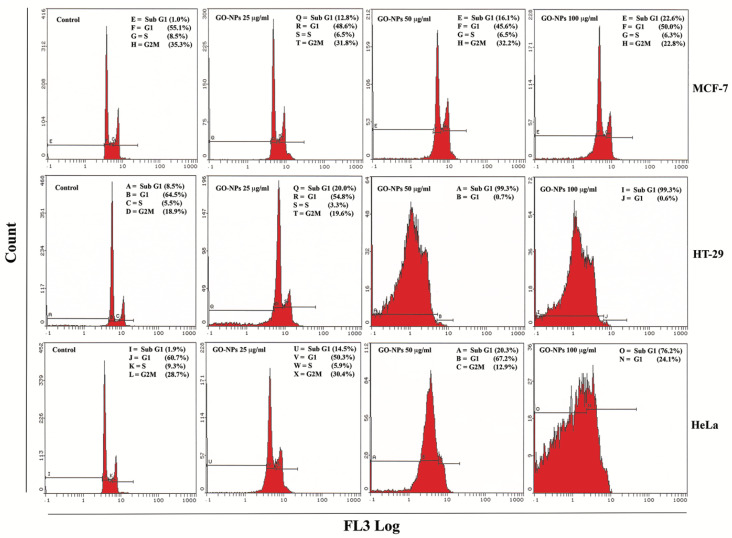
Dysfunction of the cell cycle in cancer cells analyzed using flow cytometry. Representative images of cell cycle obtained from MCF-7, HT-29, and HeLa cells displaying a greater sub-G1 peak post-GRO-NLs treatment.

GRO-NLs induced apoptosis in all three cell lines, which were quantitated using flow cytometry. Representative images of the cell cycle obtained from the GRO-NLs-treated cell lines exhibit an increase in the apoptotic (sub-G1) phase (Figure 4). Cumulative data of MCF-7 cells exposed to 25–100 μg/mL of GRO-NLs showed a 9.1%, 11.3%, and 19.1% increase in the sub-G1 phase, while the control cells showed only 1% of cells in the sub-G1 phase (Supplementary Figure S5). HT-29 cells exposed to GRO-NLs at 25, 50, and 100 μg/mL showed 16.7%, 99.3%, and 99.6% of cells in the sub-G1 phase, relatively to the 9.0% of cells recorded in their control cells. At 50 and 100 μg/mL, the S and G2M phases of HT-29 cells were not distinguishable and completely diminished. HeLa cells exposed to GRO-NLs at 25–100 μg/mL showed 12.2%, 21.1%, and 71.6% of cells in the sub-G1 phase, while the control showed 1.9% of cells in this phase (Supplementary Figure S5).

### 2.4. Oxidative Stress Quantification

**Figure 5:**
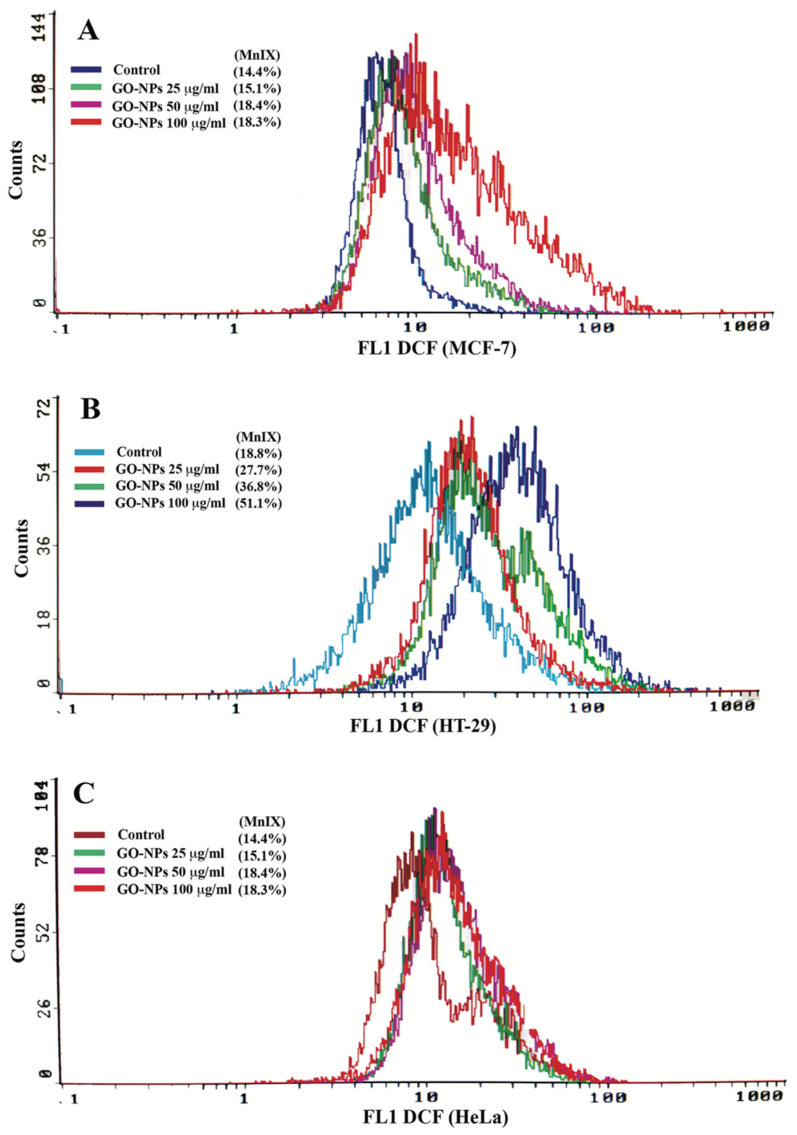
ROS quantification in cancer cells after GRO-NLs exposure. Illustrative flow cytometric overlay spectra of DCF fluorescence recorded in GRO-NLs-treated (**A**) MCF-7, (**B**) HT-29, and (**C**) HeLa cells display a greater shift on the logarithmic scale, thus affirming elevated ROS levels. MnIX indicates mean intensity of DCF fluorescence.

The three cell lines exposed to GRO-NLs (25–100 μg/mL) exhibit a logarithmic shift in DCF fluorescence (MnIX) toward greater values, as shown in the typical flow cytometric overlay images (Figure 5A–C). Relative to the control, the cumulative value showed a significant increase of 114.4% and 117.7% in DCF fluorescence in MCF-7 cells exposed to 50 and 100 μg/mL of GRO-NLs, respectively (Supplementary Figure S6). HT-29 cells exposed to 25–100 μg/mL of GRO-NLs demonstrated higher fluorescence of DCF measured at 145.5%, 201.2%, and 277.3%, relative to the control cells. HeLa cells treated with 25–100 μg/mL of GRO-NLs demonstrated 141.1%, 182.2%, and 327.2% greater DCF fluorescence (Supplementary Figure S6).

### 2.5. Mitochondrial Dysfunction Measurement

**Figure 6:**
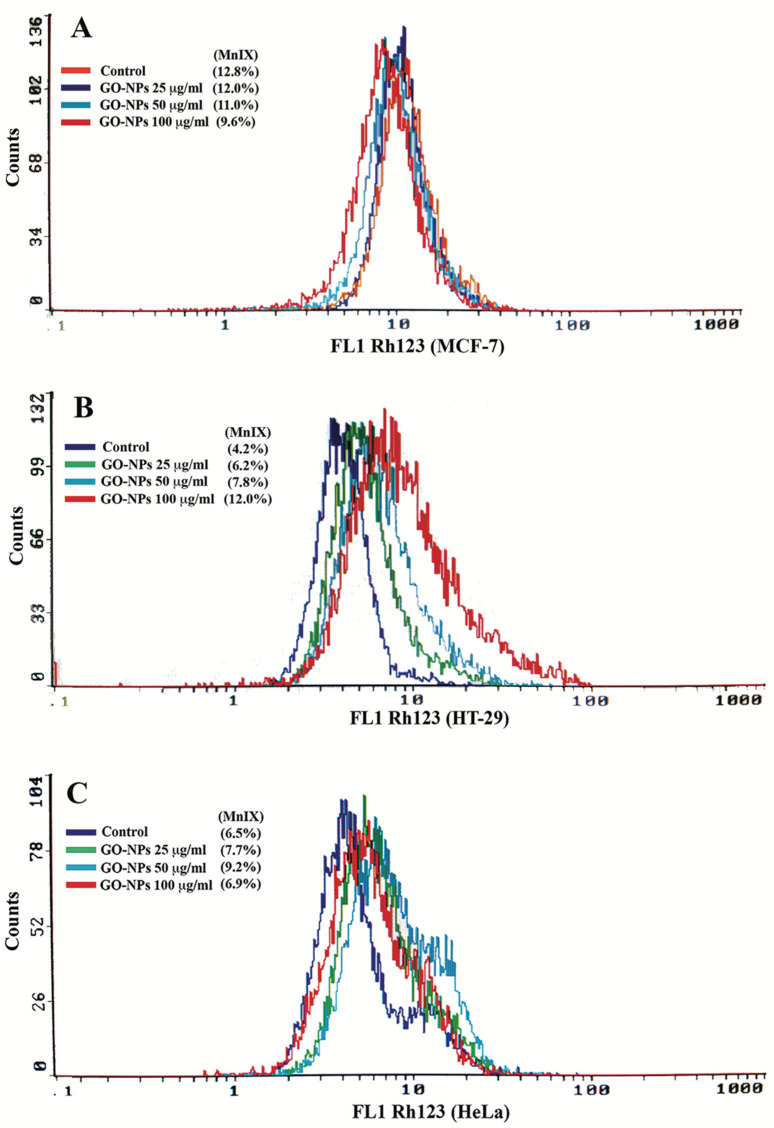
Mitochondrial membrane potential (ΔΨm) quantification via flow cytometry. Illustrative overlay spectra of Rh123 fluorescence recorded in GRO-NLs-treated (**A**) MCF-7, (**B**) HT-29, and (**C**) HeLa cells display declines and enhancements on the logarithmic scale, thus affirming mitochondrial dysfunction. MnIX indicates mean intensity of Rh123 fluorescence.

The three cell lines exposed to GRO-NLs (25–100 μg/mL) exhibit differential fluorescence of Rh123 in the flow cytometric overlay images (Figure 6A–C). MCF-7 cells exposed to GRO-NLs at 50 and 100 μg/mL exhibited 89.6% and 77.3% decline in Rh123 fluorescence, respectively (Supplementary Figure S7). HT-29 cells exposed to GRO-NLs at 50 and 100 μg/mL showed 151.3% and 250.4% higher fluorescence. HeLa cells treated with GRO-NLs (25 and 50 μg/mL) showed a significant increase of 116.8% and 146.6% (Supplementary Figure S7).

### 2.6. Induction of Ca^2+^ Influx in Cells

**Figure 7:**
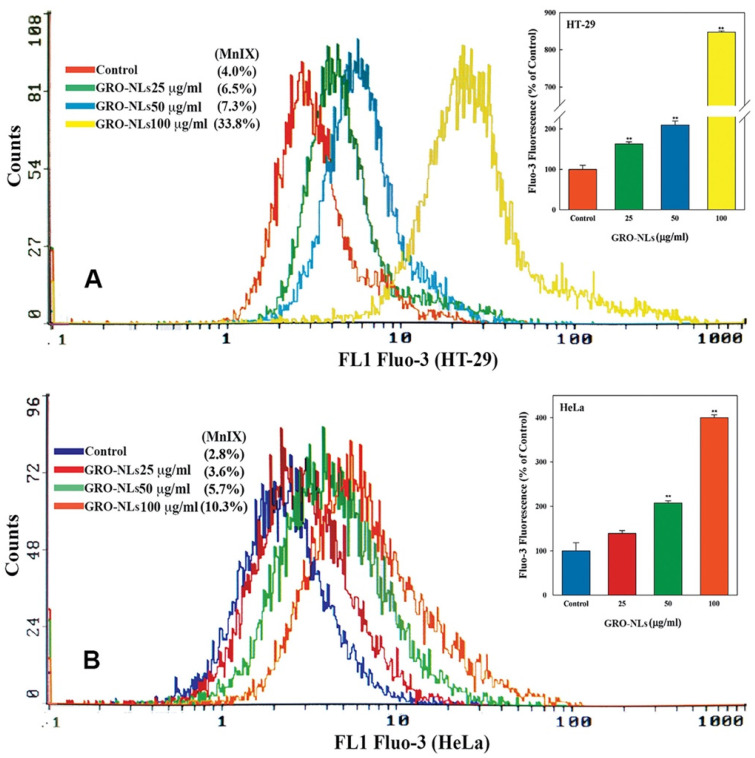
Measurement of Ca^2+^ influx in cancer cells. Illustrative fluorescence overlay spectra of Fluo-3 dye recorded in GRO-NLs-treated (**A**) HT-29 and (**B**) HeLa cells exhibit an increase in fluorescence, thus affirming the influx of Ca^2+^. MnIX indicates mean intensity of Fluo-3 fluorescence. ** p < 0.01 versus control.

The two cell lines (HT-29 and HeLa) exposed to GRO-NLs for 24 h demonstrate a massive shift of Fluo-3 fluorescence peaks toward the positive values on the log scale of the overlay graph (Figure 7A,B), while MCF-7 cells do not show any change in their fluorescence pattern. The cumulative analysis of Fluo-3 fluorescence in HT-29 cells exposed to 25–100 μg/mL of GRO-NLs showed 162.9%, 209.7%, and 847.4% greater fluorescence (Figure 7A inset). HeLa cells exposed to 25–100 μg/mL of GRO-NLs showed 138.6%, 207.4%, and 400.2% higher fluorescence of Fluo-3 relative to their control (Figure 7B inset).

### 2.7. GRO-NLs Activate Apoptotic and Oxidative Stress Genes

**Figure 8:**
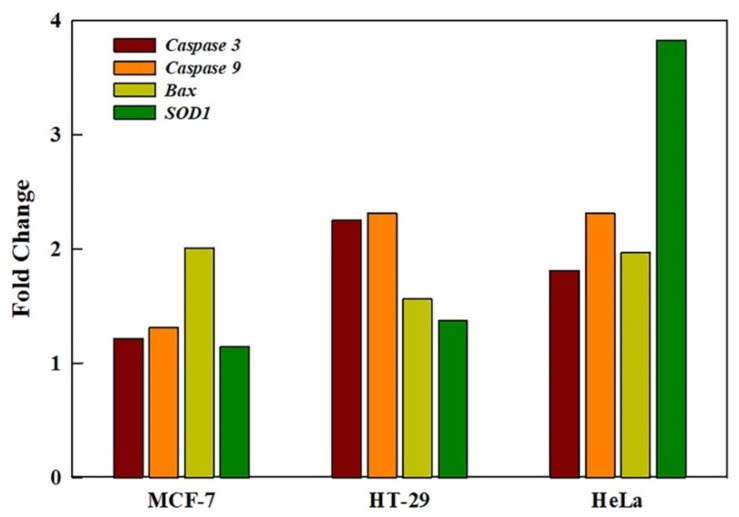
qPCR-based quantification of *caspase 3*, *caspase 9*, *bax,* and *SOD1* genes shows upregulation in three different cell lines exposed to GRO-NLs (25 μg/mL) for 24 h.

Exposure to GRO-NLs (25 μg/mL) resulted in the upregulation of the *caspase 3* gene in all three cell lines. MCF-7 cells showed a 1.21-fold increase, HT-29 cells showed a 2.25-fold increase, and HeLa cells showed a 1.81-fold higher expression of *caspase 3*. Relative to the control, *caspase 9* and *bax* were upregulated with 1.31-, 2.31-, 2.33-fold and 2.01-, 1.56-, and 1.97-fold increases in MCF-7, HT-29, and HeLa cells, respectively. *SOD1* expression in the cell lines (MCF-7, HT-29, and HeLa) was upregulated to 1.14-, 1.37-, 3.82-fold increases (Figure 8).

### 2.8. Translational Activation of Cell Cycle Proteins

**Figure 9:**
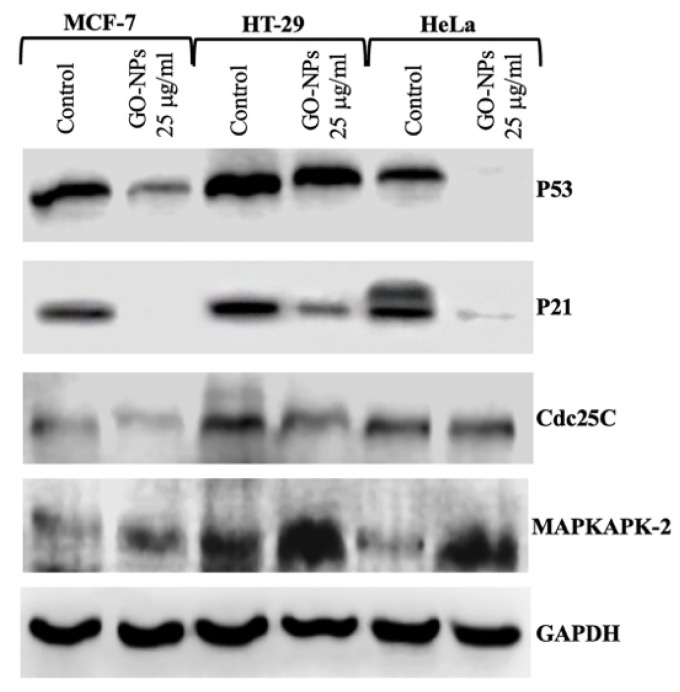
Western blot bands show the expression of cell cycle regulatory proteins in the cell lines after 24 h of GRO-NLs (25 μg/mL) treatment.

Three cell lines exposed to GRO-NLs (25 μg/mL) for 24 h exhibits activation of cell cycle regulatory proteins. Exposure of MCF-7 cells to GRO-NLs causes a depletion of P53 protein, resulting in the partial loss of the protein band, whereas the control lane shows a sharp band of P53. Under the same condition, HeLa cells exposed to GRO-NLs exhibit a complete loss of P53 protein, compared to the conspicuous band of P53 protein in its control lane. HT-29 cells exposed to GRO-NLs exhibit a slight depletion of P53 protein compared to the control band of P53 protein (Figure 9). Relative to the control band of P21 protein, GRO-NLs-treated MCF-7 cells exhibit a complete loss of band, HeLa cells show a partial loss of the P21 protein band, and HT-29 cells show a depletion of P21 protein (Figure 9). The expression of Cdc25C protein in the GRO-NLs-treated cell lines (MCF-7, HT-29, HeLa) was depleted. Expression of the MAPKAPK2 protein in the GRO-NL-treated cell lines was overexpressed, as compared to the corresponding band in the control lane (Figure 9). The band calculation of P53, P21, Cdc25C, and MAPKAPK-2 is shown in Supplementary Figures S8–S11.

## 3. Discussion

Surface modification or functionalization of GO often requires multiple preparatory steps as well as a range of tedious chemical reactions and separations. Hence, there is a constant demand for simple, continuous, and effective methodologies for the on-demand fabrication of bio-functional GO-based nanomaterials. In such nano-dimensional configuration, cellular internalization is highly anticipated, thereby increasing the number of biomedical applications by several folds. Herein, we designed and prepared a novel system called nonfunctionalized graphene oxide nanolayers (GRO-NLs). 

We provide the first evidence that GRO-NLs, behave as an anticancer entity in three independent cancer cell lines. In this study, we synthesized GRO-NLs from pristine graphite, which was confirmed using PXRD. The appearance of fingerprint broad reflection on the PXRD patterns of the GRO-NLs and the appearance of graphite feature reflection at 26.6° clearly implicate the ample oxidation of graphite on the GRO-NLs and the appearance of oxidative carrying functionalities on the GRO-NLs surfaces [41,42]. When graphite was oxidized to GRO-NLs, this interlayer distance increased to 6.51 Å, which may be ascribed to the inclusion of oxygen-possessing groups between the graphite sheets during the oxidation process [43]. On the other side, the GRO-NLs thermal degradation performance was extremely lower than that of graphite; such a property endorses the existence of O-bearing groups on the GRO-NLs plane. Relatively speaking, the TGA curve of graphite displays the entire mass loss [44]. The thermal instability of GRO-NLs can be related to the thermal decomposition of oxygenic functionalities [45]. In addition, the weight loss of GRO-NLs could be an outcome of the pyrolysis of the carbonaceous skeleton [46]. The attained thermal data are highly consistent with previous reports [47,48]. The FTIR spectra of the GRO-NLs confirm the stretching vibration of carbonyl groups and the carbonaceous backbone from graphitic areas [49,50]. Additionally, the UV-vis data of the GRO-NLs are in accordance with a previous report [51]. A Raman spectrum is a remarkable descriptive tool not only to unravel the structures of graphene but also its derivatives. As predicted, the GRO-NLs Raman pattern is demonstrated to possess two fingerprint bands (i.e., G- and D-bands) [52]. Generally, G-bands are formed due to the well-organized graphene lattice structure of the vibrations of the sp^2^ structure, whereas D-bands are commonly associated with deficiencies or disorder in the graphene structure [53]. The morphological analysis of the GRO-NLs using HR-TEM confirm that the synthesized GRO-NLs exist in the form of transparent, highly exfoliated nanolayers composed of thin flakes. 

After the successful synthesis of GRO-NLs, we focused our study on quantifying their cytotoxic outcomes in three different cancer cell lines. These cell lines were preferentially chosen to assess the broad ability of the GRO-NLs to obstruct different cancer cell types. MCF-7 is regarded as a “workhorse” and is widely exploited in in vitro studies to analyze glucocorticoids, progesterone and estrogen-positive breast cancer, and androgen receptors [54,55]. On the other hand, HT-29 cells are primarily used in colon cancer research, precisely to analyze intestinal cell differentiation. HT-29 cells show the characteristics of different human intestinal cells [56]. HeLa cells are a popular choice for studies on the human epithelium. In particular, apoptosis in human cells occurs when drugs or anticancer agents come in contact with them while entering through the epithelium [57,58]. The GRO-NLs-treated cancer cells exhibited morphological changes observed as gap formation among adjacent cells, cell shrinkage, and cellular detachment from the culture plate that emerged as floating round cells in the culture medium. Such changes were more pronounced in the HT-29 and HeLa cells, while the MCF-7 cells showed less detachment. The survival of all cell lines was significantly decreased after GRO-NLs exposure, as evident by the MTT assay data. Quantitative analysis based on the MTT assay exhibited maximum effect on the survival of HT-29, HeLa, and MCF-7 cells. Our MTT data are in line with the cytotoxic effects of multilayer GO in HaCaT cells [59]. The activities of cellular dehydrogenases and the MTT assay classically rely on each other, the former being chiefly confined in mitochondria for the reduction of yellow dye to formazan, a strong light-absorbing end-product. Variable MTT assay responses of the three cell lines in our study epitomize the influence of GRO-NLs on mitochondrial depolarization effects, which restrain the stockpiling of dye in mitochondria [60,61]. Moreover, the GRO-NLs were tested for their cytotoxic effects by measuring the lysosomal functionality in cancer cells. Relative to the MTT data, in the NRU assay, we found greater cytotoxic effects induced by the GRO-NLs. As such, HT-29 and HeLa cell survival was maximally decreased by GRO-NLs treatment, while MCF-7 was least affected. The observed changes in lysosomal function imply a disintegration of the delicate lysosomal membrane in the cancer cells, which leads to instability and abrogation of neutral dye uptake via sequestration of lysosomal acid phosphatase [62]. In addition, vulnerability of lysosomes is envisaged as an a priori incident of damage in mitochondria [63]. To a larger extent, our cytotoxicity data on the GRO-NLs are in close agreement with some recent reports confirming the higher cytotoxic effects of GO flakes in neuronal cell lines (SH-SY5Y and HEK-293) and human colon cancer cells (HCT116), especially at higher concentrations [64,65].

The GRO-NLs were further evaluated for their apoptotic potential in cancer cells. The cell cycle analysis in HT-29 and HeLa cells demonstrated a greater amount of sub-G1 peak post-GRO-NLs exposure. A lower percentage of MCF-7 cells was exhibited in the sub-G1 phase after GRO-NL exposure; nonetheless, the highest treatment concentrations were significantly different from their respective control. Varying levels of apoptotic responses among the cancer cell lines could be due to differences in endocytic and phagocytic mechanisms upon the interaction of GRO-NLs with cellular membranes. A similar response had been observed earlier and was linked with the induction of apoptosis and necrosis in BEAS-2B and A549 cells after exposure to thermally reduced GO (TRGO) [66]. Moreover, we also postulate that the irregular shapes of the GRO-NLs, with greater roughness and shape edges, assist in their higher interaction with cell membranes [67]. 

GO can raise ROS levels that interfere with the functionality of mitochondrial membrane potential, which ultimately triggers apoptosis in cells. In this notion, we also found that GRO-NLs exposure produced ROS in all three cell lines, which unequivocally validates their prospects as an oxidative stressor for cancer cells. A comparable finding previously found in SH-SY5Y cells also confirmed that GO large flakes (4.9 ± 3.8 μm) and GO 1 h small flakes (151.6 ± 1.9 nm) had the propensity to enhance intracellular ROS production [64]. In the same line of work, both A549 and BEAS-2B treated with GO and thermally reduced GO (TRGO) showed a significant increase in ROS levels [64,66]. ROS are extremely reactive molecules that interact with cell organelles and membranes to prompt damage. More importantly, if there is impairment in mitochondrial functionality, which is reflected in the form of ROS accumulation. ROS has the features of a double-edged sword; at lower doses, it manages cell signaling, and at higher doses, it shows cytotoxicity [68]. At a mild level, ROS regulates indispensable cellular processes such as development and homeostasis, whereas higher levels trigger cellular damage [69,70]. Cancer cells are very susceptible to the presence of prooxidant and antioxidant inhibition owing to their higher levels of ROS [71,72,73]. There is the existence of ROS-induced killing of cancer cells, occurring upon oxidative-stress dependent cytotoxicity and cell death via apoptosis or necroptosis, and autophagy [74]. In this respect, the nonfunctionalized GRO-NLs exhibited greater ROS-generated cytotoxicity, ultimately triggering cell death via apoptosis and, thus, indicating the prospects of ROS-dependent killing of cancer cells. 

Consequently, we analyzed mitochondrial integrity in the cancer cell lines. The flow analysis showed that GRO-NLs exposure enhanced Rh123 fluorescence in HT-29 and HeLa cells, whereas MCF-7 showed fluorescence decline. Enhancement of Rh123 fluorescence in mitochondria has been associated with its inherent properties to swell and shrink depending on irresistible situations, viz., Ca^2+^ influx, cytochrome c release, and pH during apoptosis. Swelled mitochondria fail to retain the dye and leak it into the cytoplasm, causing hyperpolarization [75], whereas Rh123 fluorescence decline is linked with ΔΨm dissipation owing to disturbances in mitochondrial inner membrane permeability or proton-moving force [76]. It has been reported that Ca^2+^ flux and mitochondrial ROS production may assist a unique form of programmed necrosis [77]. The GRO-NLs-treated HT-29 and HeLa cells exhibited an upsurge in Ca^2+^ flux, as evident by the shift of the overlay spectra of Fluo-3 dye to larger values on the log scale. Mitochondria have the ability to take up Ca^2+^ for the regulation of spatiotemporal patterns of Ca^2+^ signaling, a process crucial for different cellular activities. Nevertheless, excessive uptake can trigger damage in mitochondria and enhance ROS production in them [78,79]. Predominantly, our data on GRO-NLs-induced ROS generation, ΔΨm, and Ca^2+^ influx are in close agreement with studies that have exhibited identical responses in liver and skin cells after exposure to GO [77,80]. 

We further quantitated the activation of genes pertaining to the apoptotic and oxidative stress pathways. Relative to the control, the HT-29, HeLa, and MCF-7 cells exposed to GRO-NLs demonstrated upregulation of apoptotic genes (*caspases 3*, *9*). Stimulation of these genes implicates mitochondrial impairment, owing to the fact that caspases are crucial for apoptosis. Mitochondrial membrane dysfunction (ΔΨm), as also evident in the flow cytometric data, is a key factor in triggering the cascade of the mitochondrial-dependent intrinsic pathway. Therefore, cytochrome c is released to activate caspases 9 and 3 [81]. Caspase 3 is observed as a “point-of-no-return” in the multi-step signaling of apoptosis, which is clearly validated by our data exhibiting a larger percentage of apoptosis peak (sub-G1) in cancer cells after GRO-NLs treatment [82]. *Bax* was upregulated in the GRO-NLs-treated cancer cells, and this result is in accordance with an earlier study that also reported its upregulation in the liver and kidney of mice after GO and rGO treatments [83]. Upregulation of *bax* was recorded in cells that were under stress and undergoing apoptosis [84]. We found that HT-29, HeLa, and MCF-7 cells exposed to GRO-NLs showed upregulation of the oxidative stress gene (*SOD1*), clearly indicating that free radicals produced in the cells might be scavenged by the cytoplasmic SOD1. Nonetheless, excess oxidative stress, as also evident in our ROS data, was beyond the mitigating ability of SOD1 to subside the oxidative damage and rescue the cells from apoptosis. The upregulation of *bax* and *SOD1* transcripts in our study may serve as biomarker genes for GO-based molecular toxicity in cells. 

We found intriguing responses in the Western blotting experiments. After the GRO-NLs treatment, P53 protein expression was depleted in HT-29 and MCF-7 cells, while it was abolished in HeLa cells. Furthermore, P21 protein (P21^CIP1^ or P21^WAF1^) expression was also depleted in HT-29 cells, more or less abolished in HeLa cells, and eliminated in MCF-7 cells. Being a tumor suppressor protein, P53 activation is interconnected with the upregulation of P21 protein [85]. P53 protein is capable of transactivating three downstream genes, including P21, which acts as a main downstream effector of P53 to mediate G1/S arrest [86,87]. A strong association between DNA damage, accumulation of P53 protein, and P21 expression after exposure to DNA-damaging agents has been reported [86,88,89,90,91]. Notwithstanding the fact that P21 protein expression has been noticed in wild-type P53, cells deficient in P53 protein activity do not show P21 expression. It has been validated that mutations in the P53 gene can lead to dysfunction in P53 protein with respect to its ability to induce P21 expression [92]. Hence, in this analogy, it can be envisaged that the GRO-NLs might have acted as a mutagen to induce mutations in the P53 gene, leading to the dysfunction of P53 protein in the three cancer cell lines. Abnormalities in P53 protein subsequently altered the downstream effector protein P21 in the cancer cell lines, as also evident in earlier studies [88,91,92]. Another possibility could be mutations outside P53 or the existence of mechanisms other than P53 mutation that may control the dysfunction of P53 protein with respect to its capability to induce P21 expression. Our data are supported by the similar responses reported in a previous study [92]. We postulate that there is a possibly role for a P53-independent pathway to induce P53 mutation, which leads to the abolishment of its downstream effector P21 protein in cancer cells to cause cell death. Similar to our findings, a recent study also validated the involvement of a P53 independent pathway and abolishment of P53 protein expression in cancer cells after treatment with ZnO nanoparticles [22]. The HeLa control cells showed two bands of P21. We have no firm answer for the appearance of these two bands; nonetheless, they might have been an outcome of the phosphorylation of P21, which led to slower migration of the corresponding band [93]. 

We also found substantial depletion of Cdc25C protein in the three cell lines after treatment with GRO-NLs. Cdc25C, a dual-specificity phosphatase, plays a crucial role in balancing the cell cycle serine/threonine kinase action. Being in charge, Cdc25C promotes and maintains activation of cyclin B1/CDK1, thereby determining the checkpoint at the G2 phase of the cell cycle [94]. CHK1, CHK2, and P53 have been shown to phosphorylate Cdc25C, as well as obstruct its activity during DNA damage, resulting in its breakdown in the cytoplasm [95]. This sequence of events averts the stimulation of cyclin B1/CDK1, ultimately arresting the cells in the G2/M phase. Inhibition of Cdc25C inhibits the stimulation of downstream signaling pathways, including P21, which is required for entry into the G2M phase [96]. Moreover, the P21-encoded protein acts as an inhibitor of CDKs that binds to other cyclin (E/CDK2, D/CDK4) complexes, which leads to G1 arrest [97]. Simultaneously, cyclin B1, 14-3-3σ, and GADD45 are downstream effectors implicated in G2/M arrest. Apart from that, the translational activation of MAPKAPK2 in cancer cells after GRO-NLs exposure suggests its putative role in the phosphorylation of Cdc25C at Ser216, which assists in the cytoplasmic degradation, inactivation, and growth inhibition of cells [98]. Cell cycle checkpoints are the key regulatory mechanisms for controlling cellular obligatory processes, including DNA replication, repair, and mitosis. Nevertheless, harmful stress encourages stimulation of checkpoint proteins, arresting cells in time; if that fails, the cells enter apoptosis [99]. 

## 4. Materials and Methods

### 4.1. Synthesis and Characterization of Graphene Oxide Nanolayers (GRO-NLs)

#### 4.1.1. GRO-NL Synthesis

Graphene oxide nanolayers (GRO-NLs) were fabricated from pristine graphite powder following the modified Hummers procedure [100]. Graphite powder (0.5 g) (99.999%, 200 mesh) was added to NaNO_3_ (0.5 g), and then H_2_SO_4_ (23 mL) was gently mixed in by placing the solution in an ice bath. Subsequently, KMnO_4_ (3 g) was added into the tube and kept in a water bath (35 °C). The solution was stirred for 1 h to form a thick, dark green paste. Afterward, deionized water (40 mL) was added slowly, and additional stirring for 30 min at 98 °C was performed. Eventually, deionized water (100 mL) and H_2_O_2_ (30%) (12 mL) were dispensed in the tube, which ultimately changed the color (from dark brown to yellowish-pale brown). The solution was vacuum filtered (0.45 μm) and finally subjected to washing with deionized water to reach a pH of 6.5. The final product was stored under a vacuum for drying.

#### 4.1.2. Characterization of GRO-NLs

The graphene oxide nanolayers (GRO-NLs) crystalline structure evaluation was performed using an X-ray diffractometer (Bruker D2 Phaser, Bruker, Germany). The GRO-NLs were further characterized for FT-IR spectra using a Perkin Elmer 1000 FT-IR spectrometer (Waltham, MA, USA). The GRO-NLs TGA was quantitated by heating the samples with a N_2_ flow (10 °C/min) (Perkin-Elmer TGA 7, Waltham, MA, USA). Transmission electron microscope (TEM) imaging of the GRO-NLs was conducted using a JEM 2100F, JEOL, Tokyo, Japan. GRO-NLs was further characterized by measuring Raman spectra using a Raman microscope (Renishaw, Gloucestershire, UK), equipped with excitation source of argon ion laser (514.5 nm). Data acquisition time was 20s with laser power of 8 mW at the sample.

### 4.2. Culture Condition and GRO-NLs Exposure Concentration

MCF-7, HT-29, and HeLa cells were subcultured in DMEM with high glucose (10% FBS) in a CO_2_ incubator having an ambient humidity of 95% and a temperature of 37 °C. The MTT and NRU assays showed no cytotoxic effects at lower concentrations (0.1–10 μg/mL) after 24 h (Supplementary Figures S1 and S3). Hence, the following concentrations of GRO-NLs (25, 50, and 100 μg/mL) were studied in further experiments. Additionally, short exposure times (i.e., <24 h) did not lead to cytotoxic effects. Consequently, a 24 h exposure time was retained for further studies.

### 4.3. Morphological Analysis and Cytotoxicity Assays

The morphology of cells treated with the GRO-NLs (25–100 μg/mL) for 24 h (37 °C, 5% CO_2_) was analyzed. The cell lines were observed under an inverted microscope for morphological changes, gaps between adjacent cells, and cellular detachment. For the MTT assays, MCF-7, HT-29, and HeLa cells were independently cultured for 24 h in 96-well plates [101]. The cells were exposed to the GRO-NLs by discarding the old medium from the culture wells, and a fresh medium containing 25–100 μg/mL of GRO-NLs was dispensed in the predesignated wells and kept in an incubator for 24 h. Subsequently, the MTT experiment was performed by throwing the old medium; a total of 5 mg/mL of MTT dye/well was dispensed, and the plate was kept in an incubator for 4 h. A total of 200 μL/well of DMSO was dispensed and gently mixed to read the absorbance at 550 nm. For the NRU assay, the cell lines were separately treated with 25–100 μg/mL of GRO-NLs for 24 h. The cells were then washed with PBS, and a neutral red dye (50 μg/mL/well) was added. After 3 h of incubation, washing was performed with 1% CaCl_2_ and 0.5% CH_2_O. Finally, a solution of acetic acid (1%) and ethanol (50%) was added to all wells, and the absorbance was recorded after 20 min at 550 nm [101]. 

### 4.4. Apoptosis Analysis

The cell lines were grown for 24 h in the presence of the GRO-NLs (25–100 μg/mL) in an incubator (37 °C, 5% CO_2_). After exposure, the cells were harvested and fixed in EtOH (70%). The cells were washed twice and resuspended in 500 μL of PBS containing RNase A (50 μg/mL), PI dye (50 μg/mL), and triton-X100 (0.1%). After 1 h of staining, the cell cycle of 10,000 cells was analyzed via a flow cytometer (Coulter Epics XL/Xl-MCL, Beckman Coulter, Inc., Brea, CA, USA) [76].

### 4.5. Intracellular ROS Quantification

Flow cytometric quantification of ROS was performed by exposing the cell lines to the GRO-NLs (25–100 μg/mL) for 24 h. Subsequently, the culture medium was removed to harvest the cells via trypsinization. The cell pellets were washed and resuspended in 500 μL of PBS, to which 5 μM of DCFH-DA dye was added. The cells were kept in an incubator for 1 h; later, any variations in the dye fluorescence were measured in 10,000 cells using a flow cytometer [76].

### 4.6. Mitochondrial Dysfunction

The effects of the GRO-NLs on the mitochondrial membrane potential (ΔΨm) of cells were quantitated based on our earlier method [102]. The overnight grown cell lines were exposed to the GRO-NLs (25–100 μg/mL) for 24 h (37 °C, 5% CO_2_). The cells were trypsinized, and the pellets were resuspended in 500 μL of PBS containing Rh123 dye (5 μg/mL). Staining was performed for 1 h in an incubator; afterward, Rh123 fluorescence changes were measured in 10,000 cells using a flow cytometer.

### 4.7. Ca^2+^ Influx

The cell lines exposed to GRO-NLs (25–100 μg/mL) for 24 h were harvested, washed, and resuspended in 500 μL of PBS. Each tube was then added with a fluorescent probe, Fluo-3 (4 μM), and left in an incubator (1 h, 5% CO_2_). Finally, fluorescence variations in 10,000 cells were recorded using a flow cytometer [103].

### 4.8. qPCR Analysis

The cell lines were exposed to the lowest concentration (25 μg/mL) of GRO-NLs for 24 h in an incubator. After exposure, the cells were processed for the isolation of total RNA (RNeasy Mini Kit, QIAGEN GmbH, Hilden, Germany). The purity of RNA was determined using a nanodrop. Synthesis of cDNA was performed using lyophilized beads following the manufacturer’s instructions (GE Health Care, Buckinghamshire, UK). The details of the primers are as follows: *SOD1* (F) 5′-AGGGCATCATCAATTTCGAG-3′, (R) 5′-TGCCTCTCTTCATCCTTTGG-3′; *caspase 9* (F) 5′-CCAGAGATTCGCAAACCAGAGG-3′, (R) 5′-GAGCACCGACATCACCAAATCC-3′; *caspase 3* (F) 5′-ACATGGCGTGTCATAAAATACC-3′, (R) 5′-CACAAAGCGACTGGATGAAC-3′; *bax* (F) 5′-TGCTTCAGGGTTTCATCCAG-3′, (R) 5′-GGCGGCAATCATCCTCTG-3′; and *GAPDH* (F) 5′-CCACTCCTCCACCTTTGAC-3′, (R) 5′-ACCCTGTTGCTGTAGCCA-3′. qPCR analysis was conducted using a LightCycler^®^ 480 (Roche Diagnostics, Rotkreuz, Switzerland). The reaction volume was 20 μL, containing 10 μL of qPCR GreenMaster, 2.5 μL of cDNA (100 ng), and 7.5 μL of primers (F and R). The cycling conditions were the same as reported previously [76,104], and the fold changes of different genes were calculated based on the 2^−∆∆Ct^ method using the Cp values (Supplementary Tables S1–S3).

### 4.9. Western Blotting

The three cell lines were exposed to the GRO-NLs (25 μg/mL) for 24 h at 37 °C. Lysis was performed with a lysis buffer (1× SDS; 62.5 mM of tris-HCl; 10% glycerol, 50 mM of DTT, 2% SDS, phosphatase, and protease inhibitors). Protein determination was performed using the Total Protein Kit, Micro (Sigma Aldrich, St. Louis, MO, USA). SDS-PAGE (10%) was used for resolving an equal amount of protein (15 μg/well), which was transferred at 100 V to Immun-blot PVDF membranes (Bio-Rad, Hercules, CA, USA). Non-fatty milk (5%) was used for blocking the membranes, and washing was performed using PBST (0.1%). The primary anti-bodies (1:500) of anti-p53, anti-p21, anti-Cdc25A, and anti-MAPKPK2 were used to incubate the membranes. Anti-GAPDH was used as a loading control. The secondary antibody used for p-53, p-21, MAPKPK2, and GAPDH was goat anti-rabbit-HRP (Cat. NO. sc-6243) at a dilution of 1:5000. m-IgGκ BP-HRP (1:5000) was the secondary antibody for Cdc25A. Protein bands were visualized using C-Digit (Licor, Houston, TX, USA) [60] and Western blot band calculations were performed using the ImageJ software (v 1.53t) [105].

### 4.10. Statistical Analysis

The significance of the data was analyzed first for normality using the Kolmogorov–Smirnov test, followed by analysis of homogeneity based on Bartlett’s test of variance. One-way ANOVA was then conducted with Dunnett’s test for multiple comparisons using Prism 9. The level of statistical significance was *p* < 0.05.

## 5. Conclusions

This study provides the first evidence that nonfunctionalized GO in the form of nanolayers, i.e., GRO-NLs, exhibits its tendency to act as a potent anticancer entity in three different cancer cell lines. The fabricated GRO-NLs significantly reduced the survival of cancer cells in the order of HT-29>HeLa>MCF-7, both by affecting mitochondrial functionality and lysosomal destabilization. The GRO-NLs acted as an oxidative stressor by elevating intracellular ROS, which simultaneously altered ΔΨm, causing Ca^2+^ influx in the above cancer cell lines. The GRO-NLs triggered apoptosis in HT-29, HeLa, and MCF-7 cells, and the result appears as a sub-G1 peak in the flow cytometric analysis. Transcriptional upregulation of *caspase 3*, *caspase 9*, *bax*, and *SOD1* genes confirms mitochondria- dependent apoptosis in the cancer cell lines. GRO-NLs exposure depleted P53, P21, and Cdc25C protein expressions. Summarizing the chain of events, it can be clearly understood that GRO-NLs may have linked mutations in the upstream *P53* gene, resulting in the depletion of P53 protein. Such depletion subsequently affects the downstream effectors P21 and Cdc25C proteins, which hamper the cell cycle checkpoints in HT-29, HeLa, and MCF-7 cells, as also evident with the absence of G1, S, and G2M arrests in the flow data, ultimately forcing the cells to enter apoptosis. Above all, there may be an existence of a mechanism other than P53 mutation that controls P53 dysfunction. Though we did not quantify DNA damage and perform a translation analysis of other checkpoint proteins from the CHK and CDK families, as well as GADD45, 14-3-3σ, Wee1, and Myt1 kinases, future studies on these checkpoint proteins can provide an in-depth understanding of the mechanism involved in the anticancer effects of GRO-NLs. Over and above that, it can also be surmised that in our in vitro experiments, the nonfunctionalized GRO-NLs qualify as a potent anticancer entity. Special attention is warranted to quantify the same consequences in retarding the growth of colon, cervical, and breast cancers in suitable animal models. 

## Data Availability

Not applicable.

## Supplementary Materials

The supporting information can be downloaded at https://www.mdpi.com/article/10.3390/ijms24119141/s1.
