# Supplementary material for: Effectiveness of Nonfunctionalized Graphene Oxide Nanolayers as Nanomedicine against Colon, Cervical, and Breast Cancer Cells
Source: Int J Mol Sci. 2023 May 23;24(11):9141. doi: 10.3390/ijms24119141 (PMC10252622; doi:10.3390/ijms24119141)
Supplement: Supplementary file 1 [file ijms-24-09141-s001.zip › ijms-2293656-supplementary.docx]

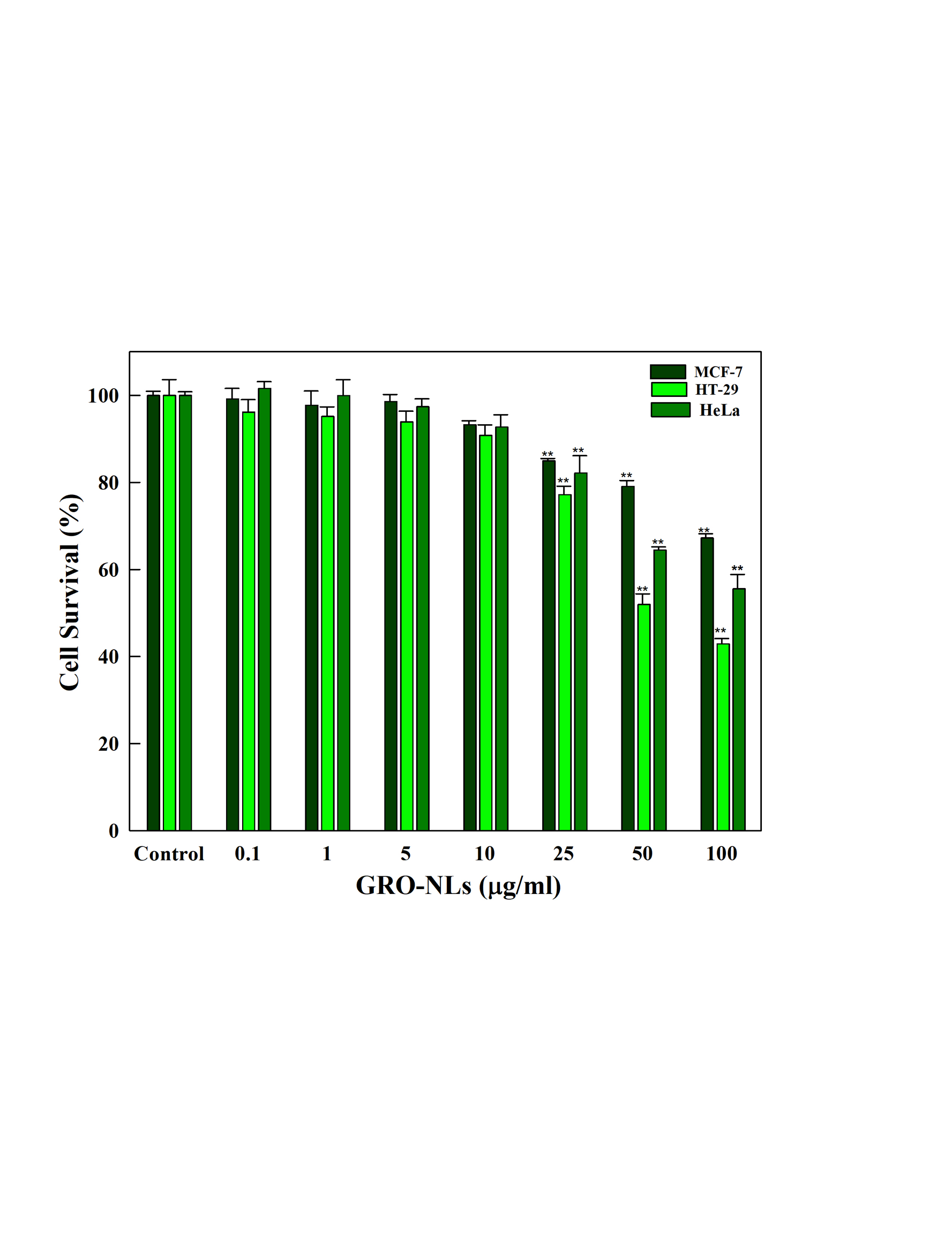


**Supplementary Figure S1:** MTT assay showing survival decline of three cell lines after GRO-NLs exposure, data are mean ± SD of 3 experiments done in triplicate wells. **p<0.01 versus control.

**
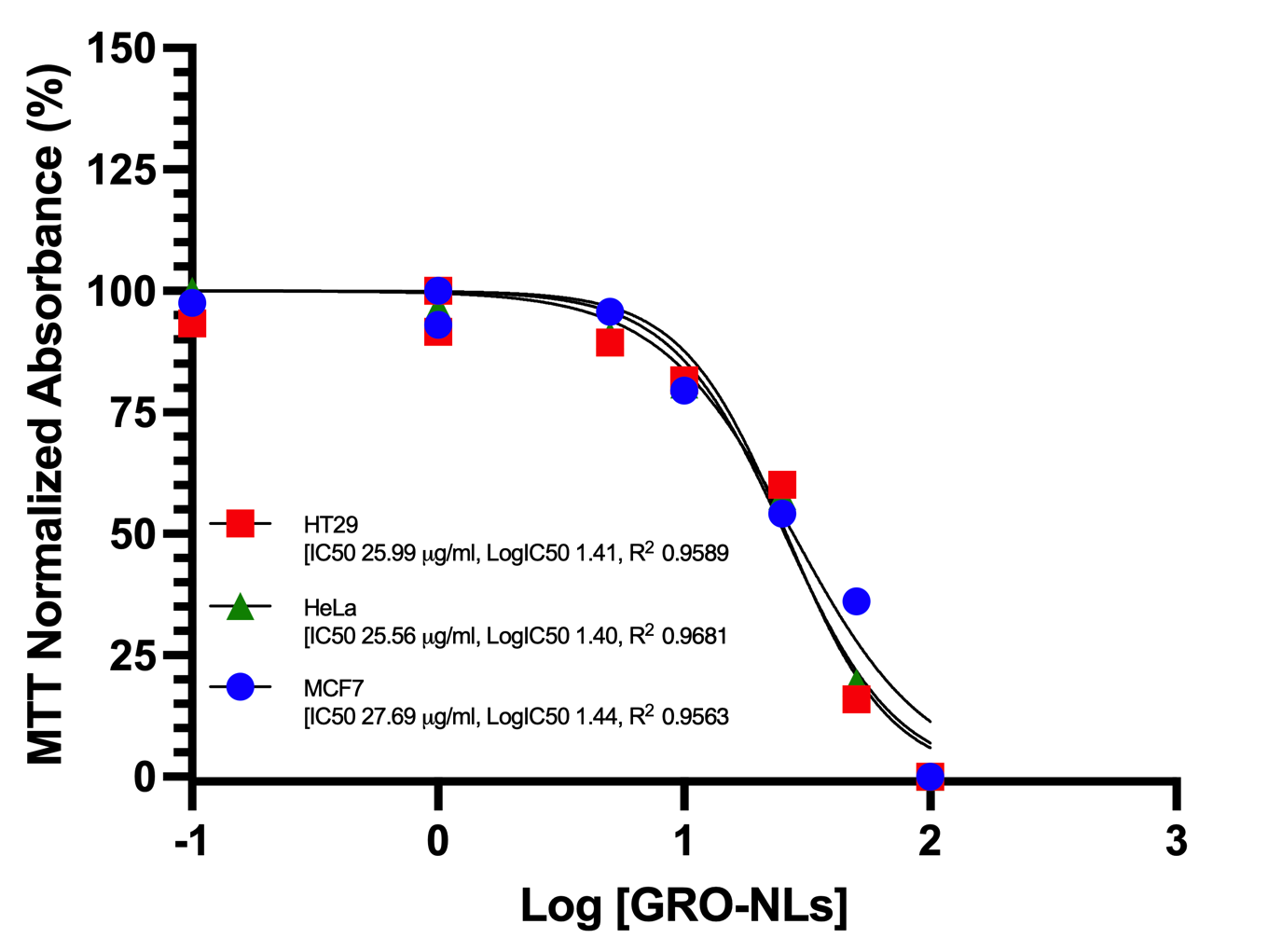
**

**Supplementary Figure S2**: GRO-NLs IC50 in MCF-7, HT29, and HeLa cells analyzed by log inhibitor versus normalized response variable slope using MTT data.


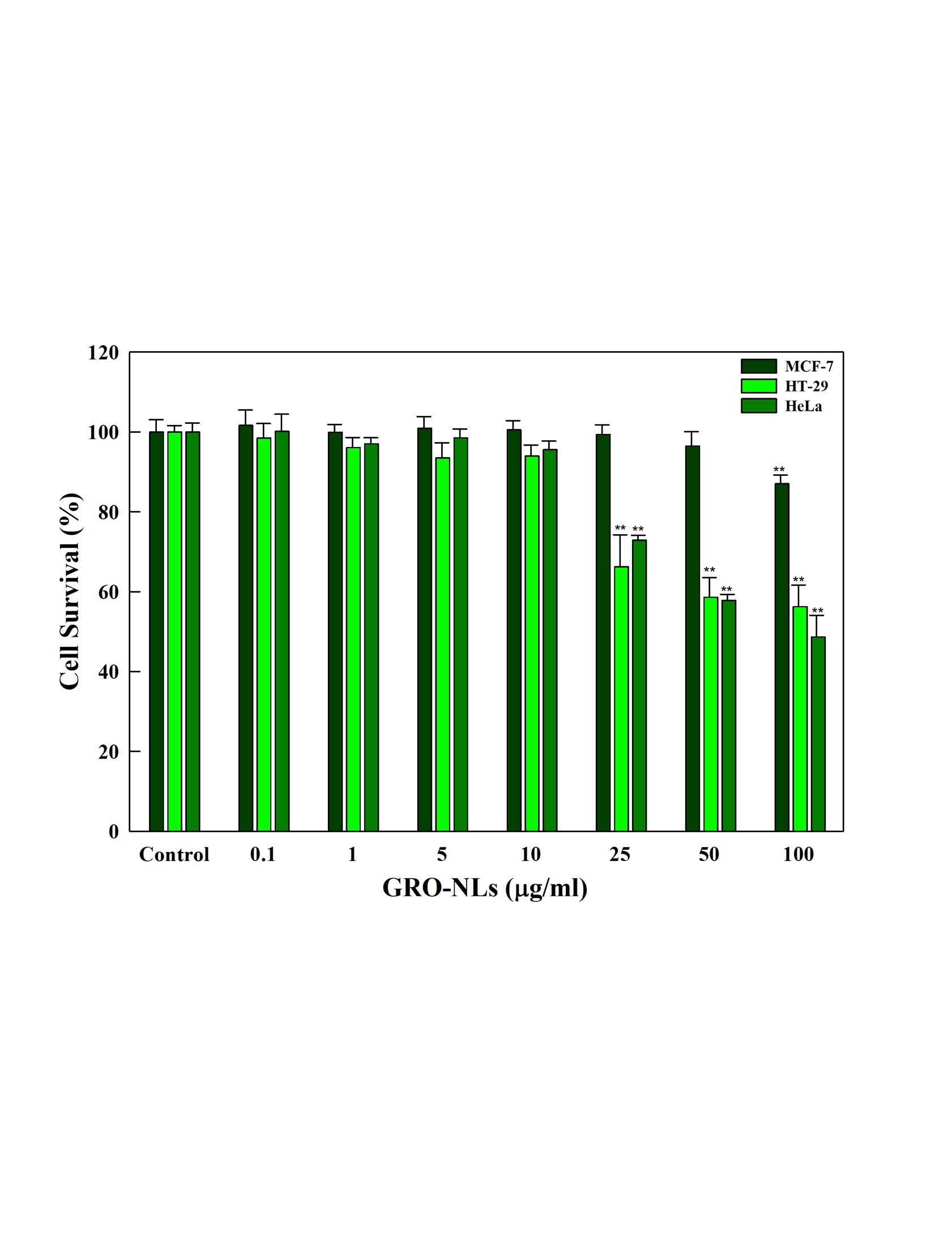


**Supplementary Figure S3:** NRU assay showing survival decline of three cell lines after GRO-NLs exposure, data are mean ± SD of 3 experiments done in triplicate wells. **p<0.01 versus control.


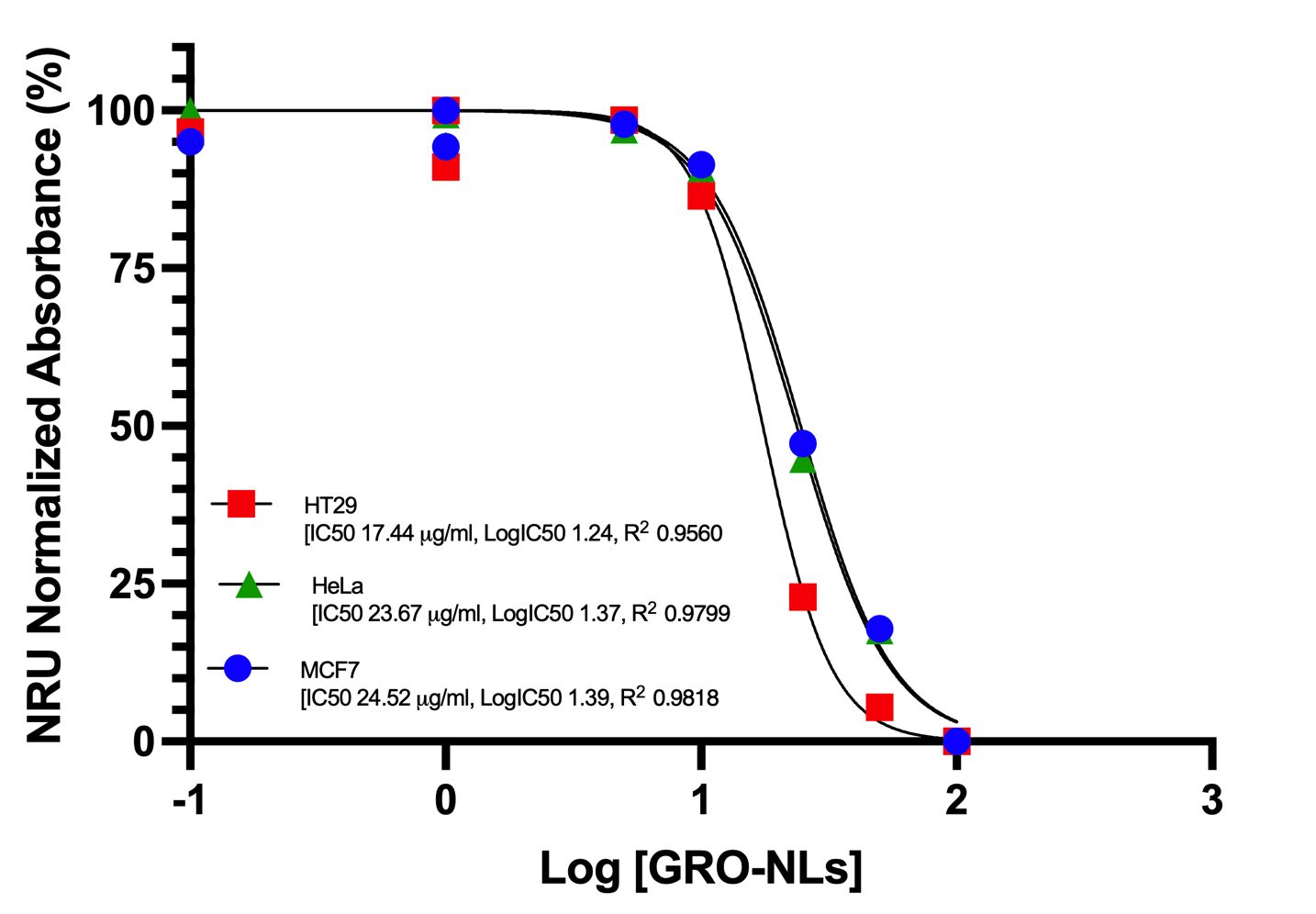


**Supplementary Figure S4**: GRO-NLs IC50 in MCF-7, HT29, and HeLa cells analyzed by log inhibitor versus normalized response variable slope using NRU data.


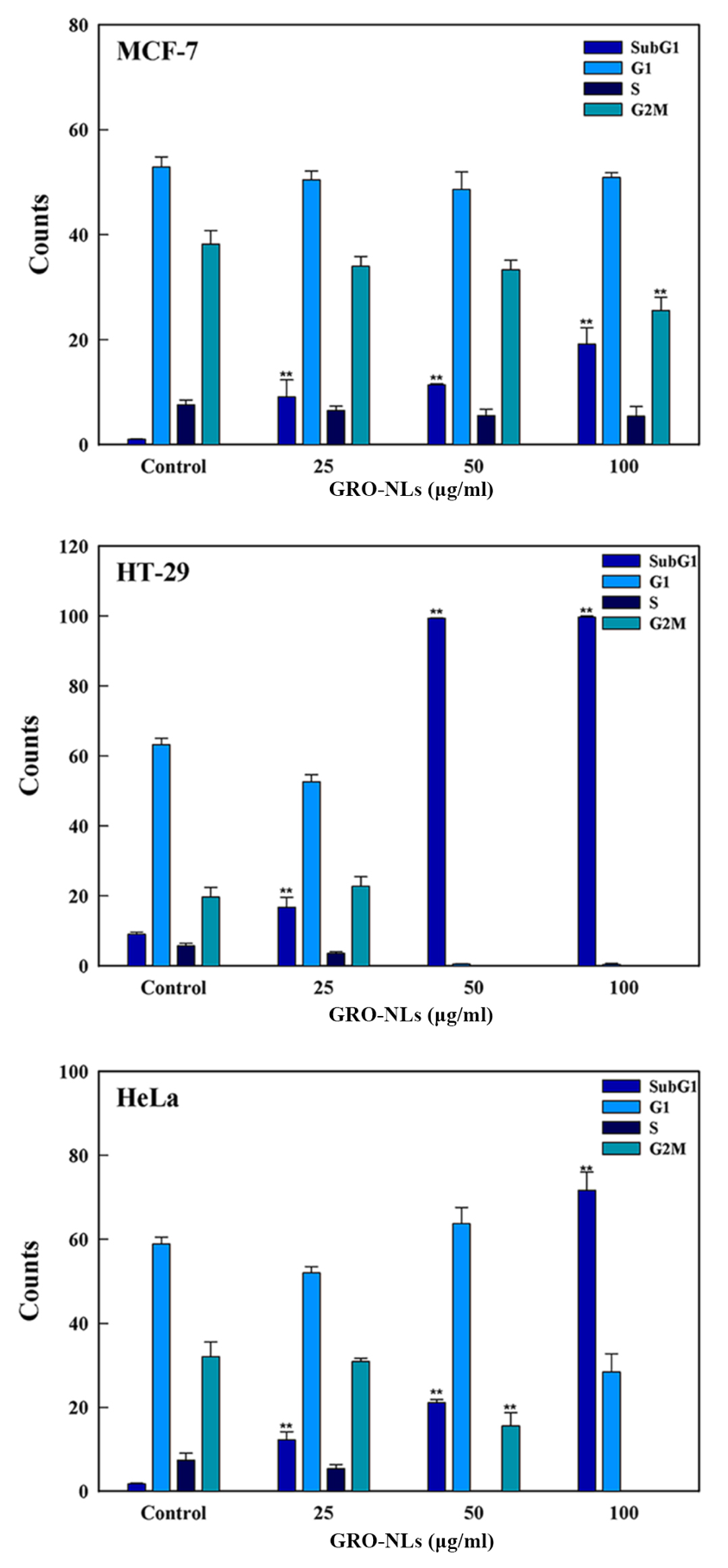


**Supplementary Figure S5:** Data in histograms are mean ± SD of varying phases of cell cycle measured after 3 experiments done in duplicated wells. **p<0.01 versus control


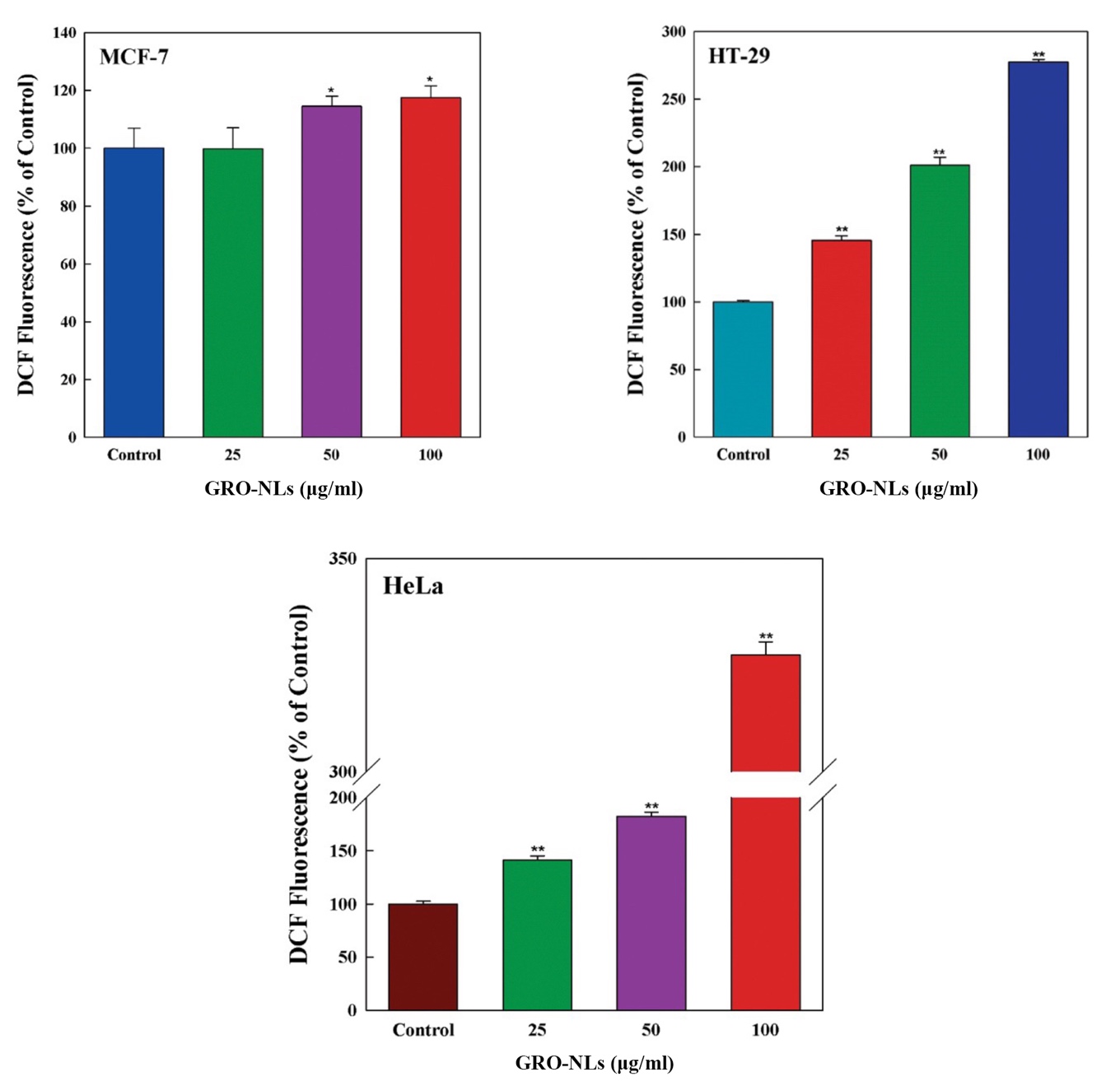


**Supplementary Figure S6:** Cumulative data of ROS generation in cancer cells after GRO-NLs exposure analyzed by measuring the MnIX (mean intensity) using a flow cytometer. **p<0.05* and ***p<0.01* verses control.


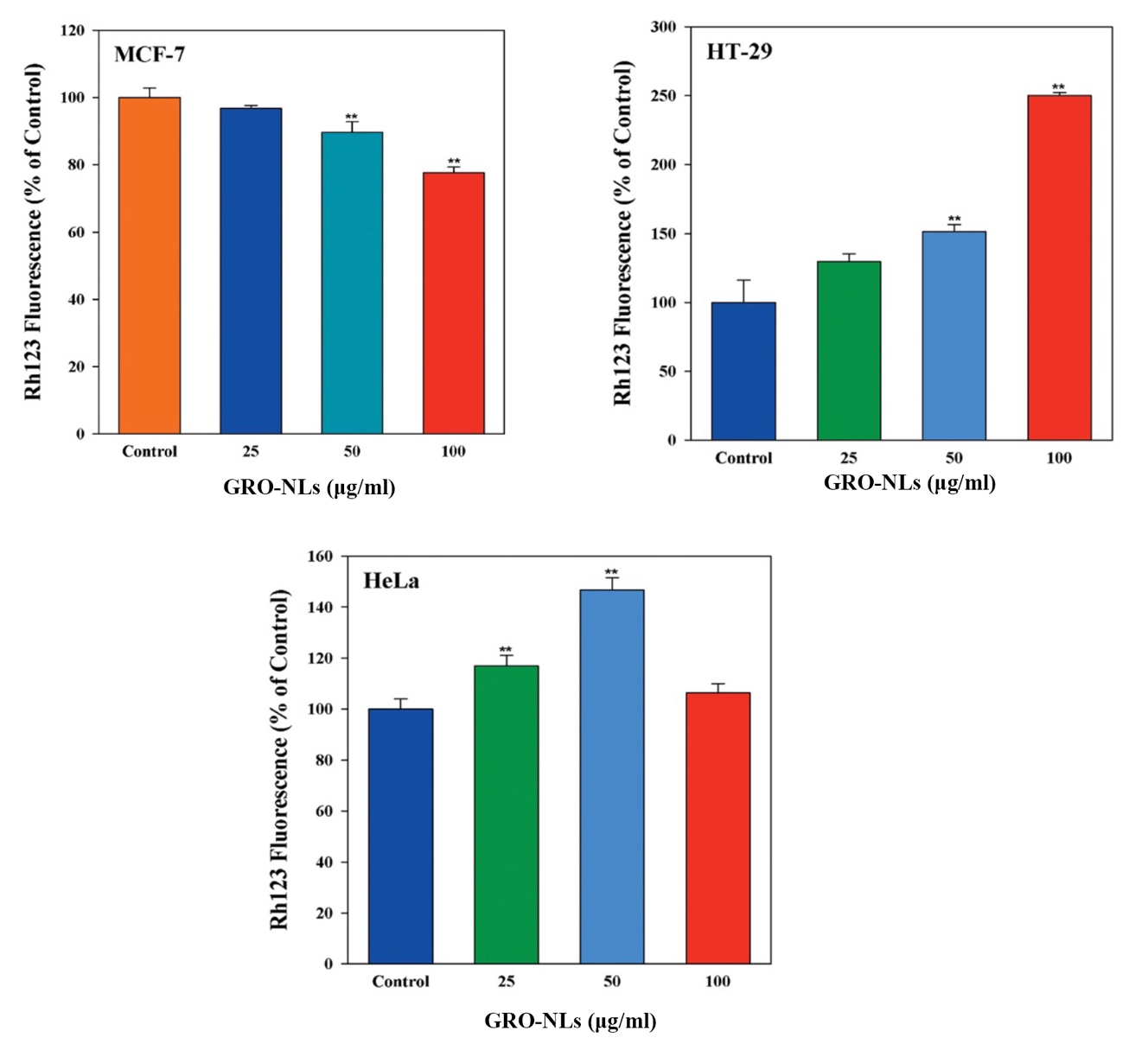


**Supplementary Figure S7:** Cumulative data of ΔΨm in cancer cells after GRO-NLs exposure analyzed by measuring the MnIX (mean intensity) using a flow cytometer. ***p<0.01* verses control.

**Supplementary Table S1.** qPCR crossing point (Cp) values of different genes in MCF-7 cells after GRO-NLs exposure. Fold change were calculated by 2^–∆∆Ct^ method.

| **MCF-7** | | | |
| --- | --- | --- | --- |
| **SOD1**  (Untreated Control) | **Cp1** | **Cp2** | **Cp3** |
|  | 27.91 | 28.83 | 28.27 |
| **SOD1**  GRO-NLs (25 μg/ml) | 28.48 | 28.08 | 27.89 |
| **HPRT**  (Untreated Control) | 31.64 | 31.48 | 31.57 |
| **HPRT**  GRO-NLs (25 μg/ml) | 31.34 | 31.49 | 31.87 |
| **Caspase 3**  (Untreated Control) | 29.77 | 29.61 | 29.34 |
| **Caspase 3**  GRO-NLs (25 μg/ml) | 29.21 | 29.31 | 29.52 |
| **HPRT**  (Untreated Control) | 31.42 | 31.68 | 31.51 |
| **HPRT**  GRO-NLs (25 μg/ml) | 31.77 | 31.38 | 31.61 |
| **Caspase 9**  (Untreated Control) | 27.78 | 27.28 | 27.31 |
| **Caspase 9**  GRO-NLs (25 μg/ml) | 27.26 | 26.87 | 27.16 |
| **HPRT**  (Untreated Control) | 31.89 | 31.34 | 31.47 |
| **HPRT**  GRO-NLs (25 μg/ml) | 31.78 | 31.45 | 31.53 |
| **Bax**  (Untreated Control) | 30.12 | 29.33 | 29.98 |
| **Bax**  GRO-NLs (25 μg/ml) | 29.13 | 28.64 | 28.81 |
| **HPRT**  (Untreated Control) | 31.62 | 31.62 | 31.62 |
| **HPRT**  GRO-NLs (25 μg/ml) | 31.68 | 31.68 | 31.68 |

**Supplementary Table S2.** qPCR crossing point (Cp) values of different genes in HT-29 cells after GRO-NLs exposure. Fold change were calculated by 2^–∆∆Ct^ method.

| **HT-29** | | | |
| --- | --- | --- | --- |
| **SOD1**  (Untreated Control) | **Cp1** | **Cp2** | **Cp3** |
|  | 31.41 | 31.12 | 31.11 |
| **SOD1**  GRO-NLs (25 μg/ml) | 31.34 | 30.66 | 30.89 |
| **HPRT**  (Untreated Control) | 31.36 | 31.13 | 31.23 |
| **HPRT**  GRO-NLs (25 μg/ml) | 31.68 | 31.39 | 31.29 |
| **Caspase 3**  (Untreated Control) | 30.73 | 31.47 | 31.16 |
| **Caspase 3**  GRO-NLs (25 μg/ml) | 29.87 | 30.49 | 30.14 |
| **HPRT**  (Untreated Control) | 31.43 | 30.97 | 31.33 |
| **HPRT**  GRO-NLs (25 μg/ml) | 31.73 | 31.28 | 31.34 |
| **Caspase 9**  (Untreated Control) | 30.95 | 30.95 | 30.95 |
| **Caspase 9**  GRO-NLs (25 μg/ml) | 30.38 | 30.38 | 30.38 |
| **HPRT**  (Untreated Control) | 31.45 | 31.45 | 31.45 |
| **HPRT**  GRO-NLs (25 μg/ml) | 31.85 | 31.85 | 31.85 |
| **Bax**  (Untreated Control) | 30.86 | 30.98 | 31.53 |
| **Bax**  GRO-NLs (25 μg/ml) | 30.65 | 30.65 | 30.65 |
| **HPRT**  (Untreated Control) | 31.58 | 30.98 | 31.18 |
| **HPRT**  GRO-NLs (25 μg/ml) | 31.43 | 31.68 | 31.24 |

**Supplementary Table S3.** qPCR crossing point (Cp) values of different genes in HeLa cells after GRO-NLs exposure. Fold change were calculated by 2^–∆∆Ct^ method.

| **HeLa** | | | |
| --- | --- | --- | --- |
| **SOD1**  (Untreated Control) | **Cp1** | **Cp2** | **Cp3** |
|  | 28.6 | 28.3 | 28.35 |
| **SOD1**  GRO-NLs (25 μg/ml) | 26.82 | 26.44 | 26.16 |
| **HPRT**  (Untreated Control) | 31.85 | 31.71 | 31.66 |
| **HPRT**  GRO-NLs (25 μg/ml) | 31.87 | 31.45 | 31.56 |
| **Caspase 3**  (Untreated Control) | 30.56 | 31.5 | 31.11 |
| **Caspase 3**  GRO-NLs (25 μg/ml) | 30.56 | 29.89 | 30.08 |
| **HPRT**  (Untreated Control) | 32.11 | 31.78 | 31.45 |
| **HPRT**  GRO-NLs (25 μg/ml) | 31.88 | 31.43 | 31.56 |
| **Caspase 9**  (Untreated Control) | 30.76 | 30.88 | 31.45 |
| **Caspase 9**  GRO-NLs (25 μg/ml) | 29.89 | 29.63 | 29.77 |
| **HPRT**  (Untreated Control) | 31.94 | 31.68 | 31.59 |
| **HPRT**  GRO-NLs (25 μg/ml) | 31.68 | 31.73 | 31.58 |
| **Bax**  (Untreated Control) | 31.56 | 30.61 | 30.73 |
| **Bax**  GRO-NLs (25 μg/ml) | 29.91 | 30.12 | 29.39 |
| **HPRT**  (Untreated Control) | 31.98 | 31.34 | 31.89 |
| **HPRT**  GRO-NLs (25 μg/ml) | 31.78 | 31.49 | 31.56 |


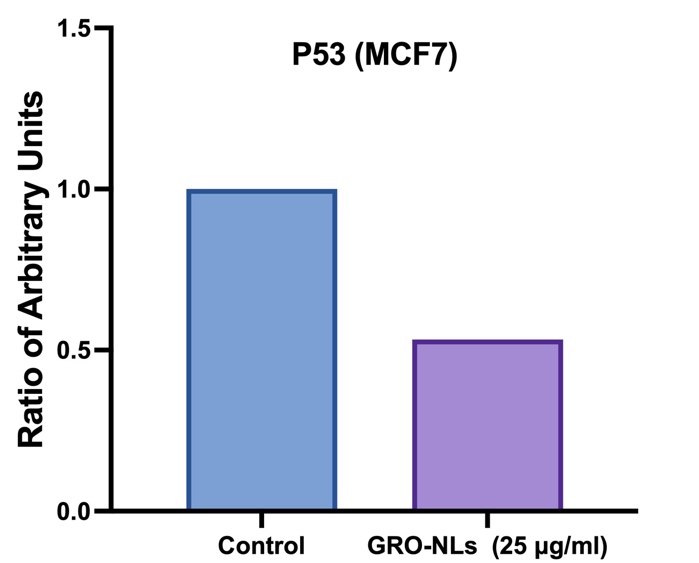


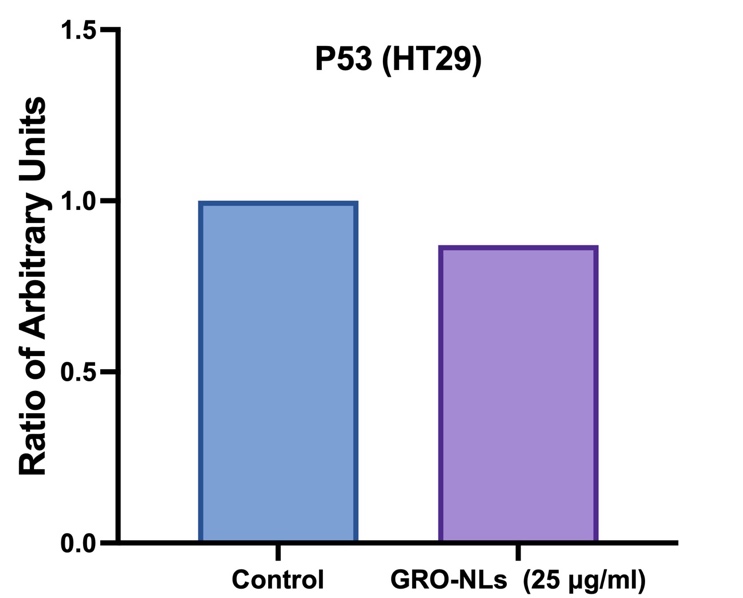


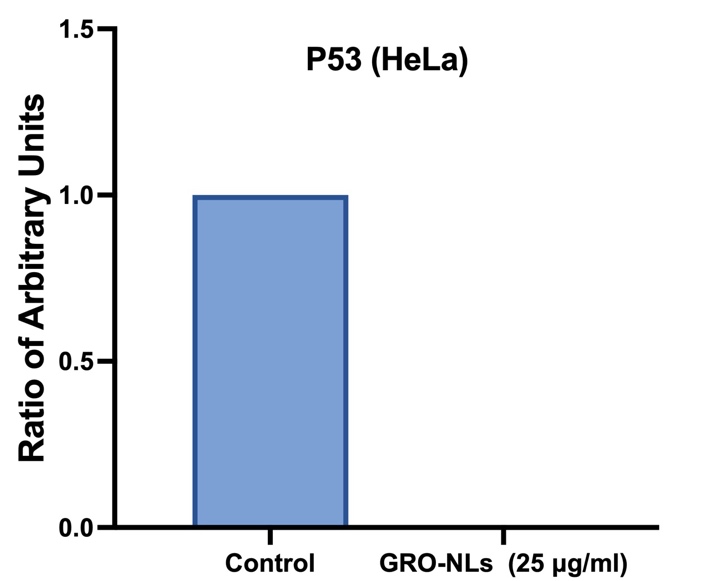


**Supplementary Figure S8**: Western blot band calculation of P53 expression in MCF-7, HT29, and HeLa cells by Image J software (https://imagej.nih.gov/ij/download.html)..


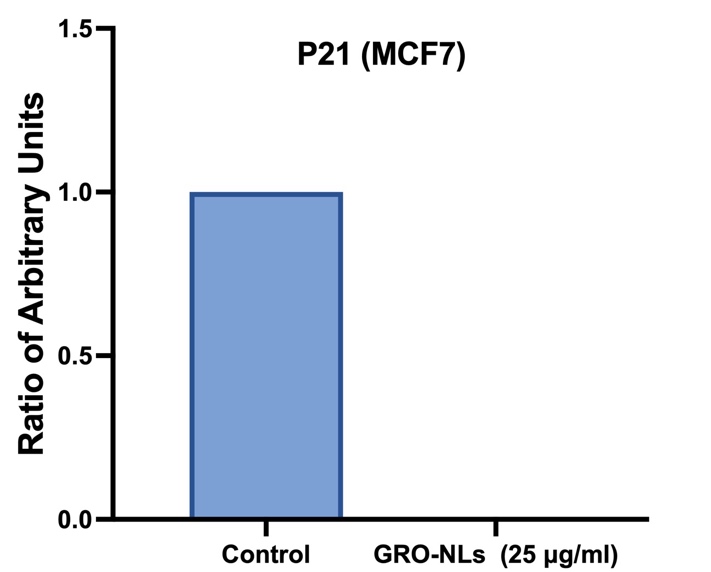


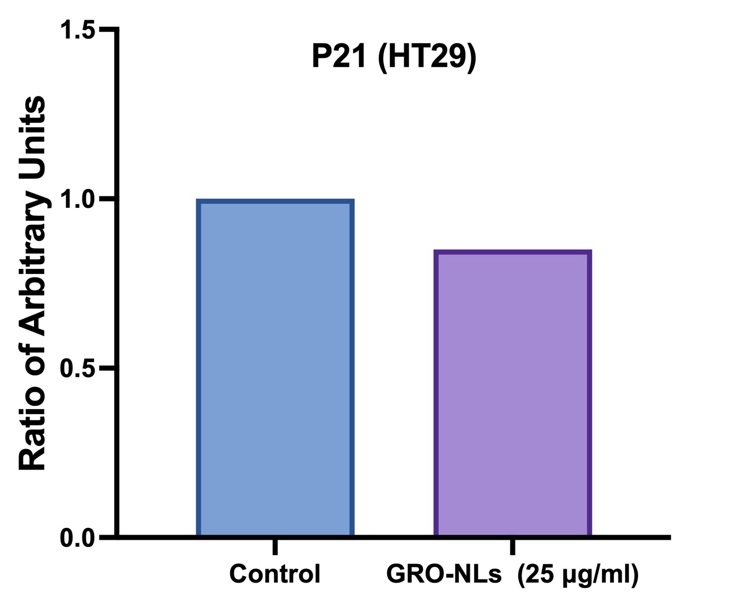


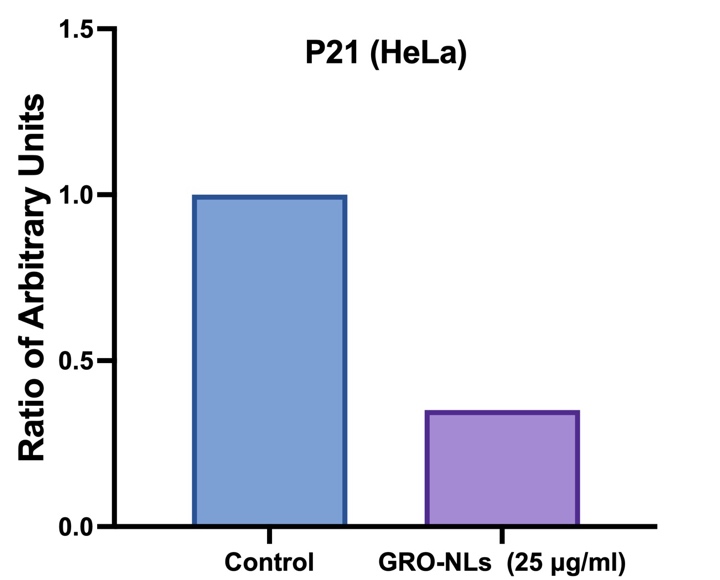


**Supplementary Figure S9**: Western blot band calculation of P21 expression in MCF-7, HT29, and HeLa cells by Image J software (https://imagej.nih.gov/ij/download.html).


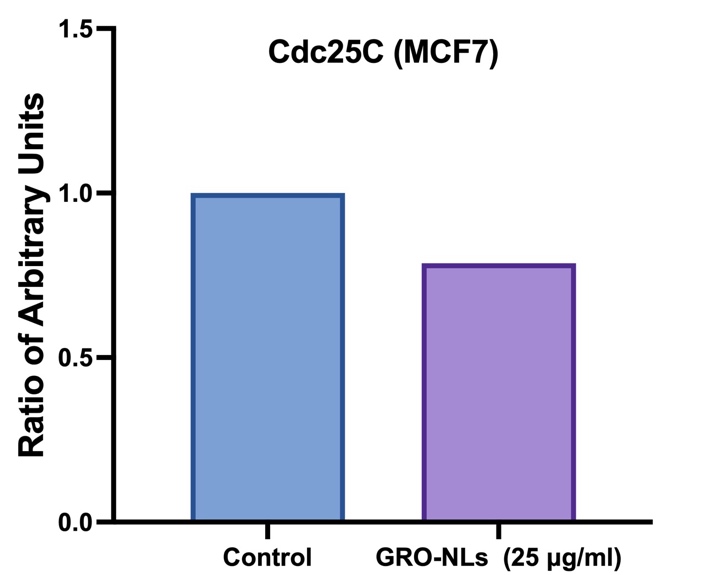


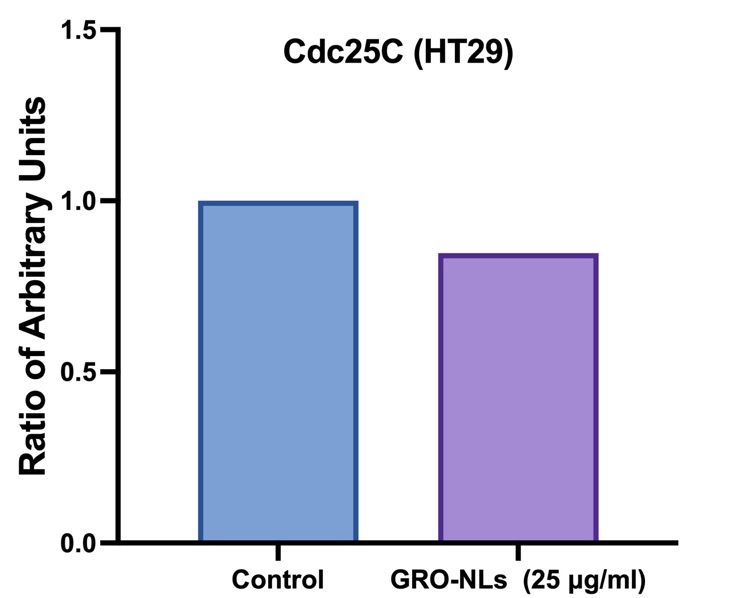


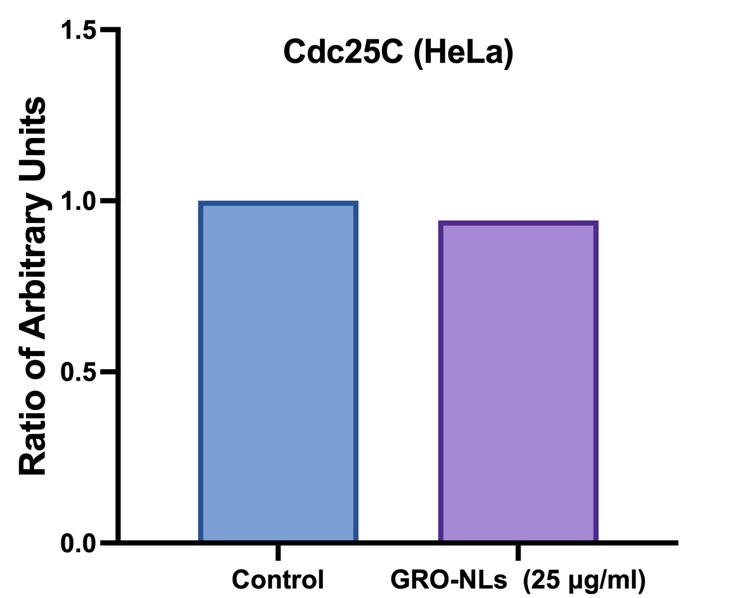


**Supplementary Figure S10**: Western blot band calculation of Cdc25C expression in MCF-7, HT29, and HeLa cells by Image J software (https://imagej.nih.gov/ij/download.html).


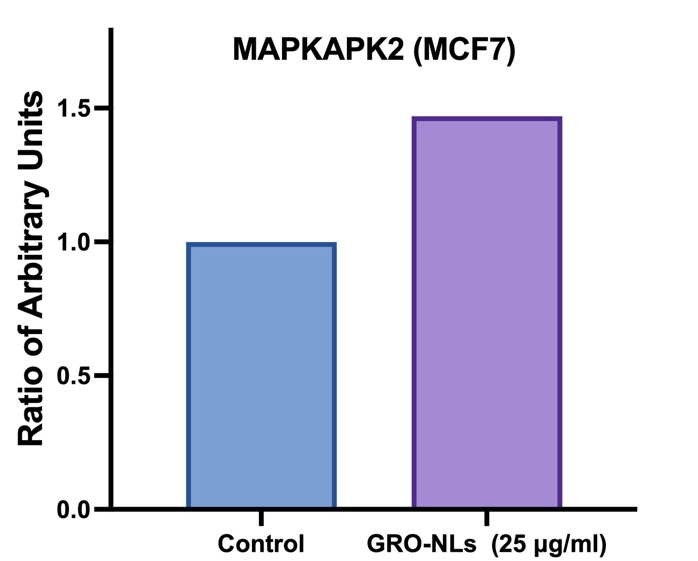


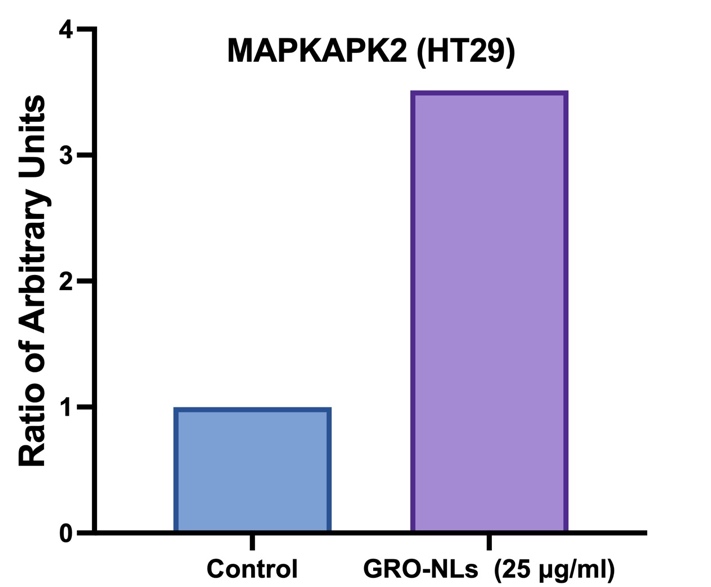


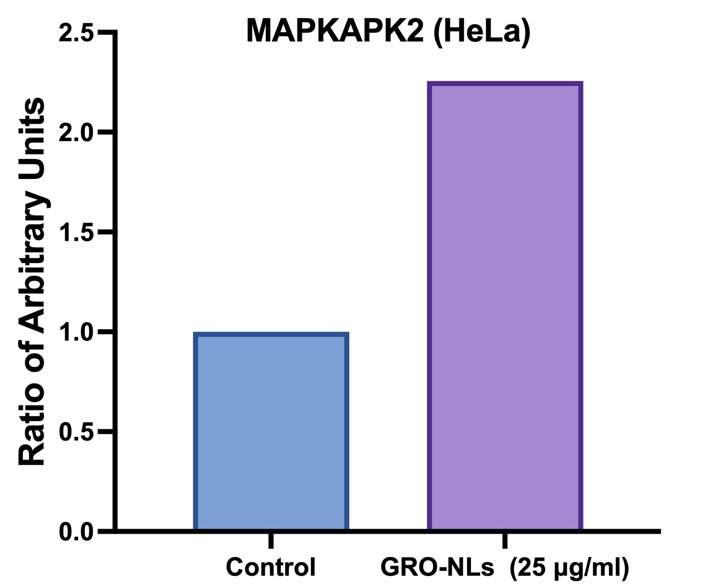


**Supplementary Figure S11**: Western blot band calculation of MAPKAPK2 expression in MCF-7, HT29, and HeLa cells by Image J software (https://imagej.nih.gov/ij/download.html).


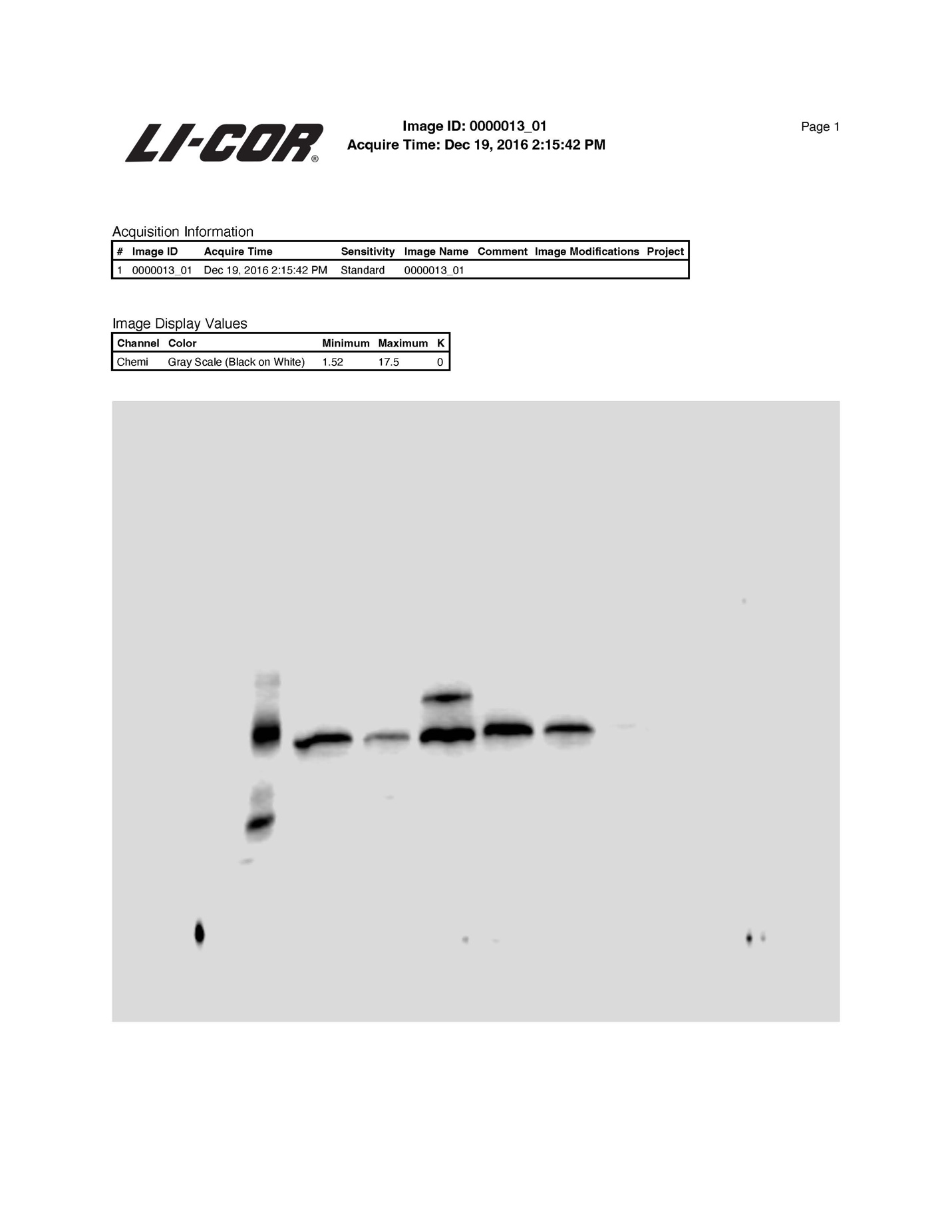


P53 western blot data (Uncropped image).


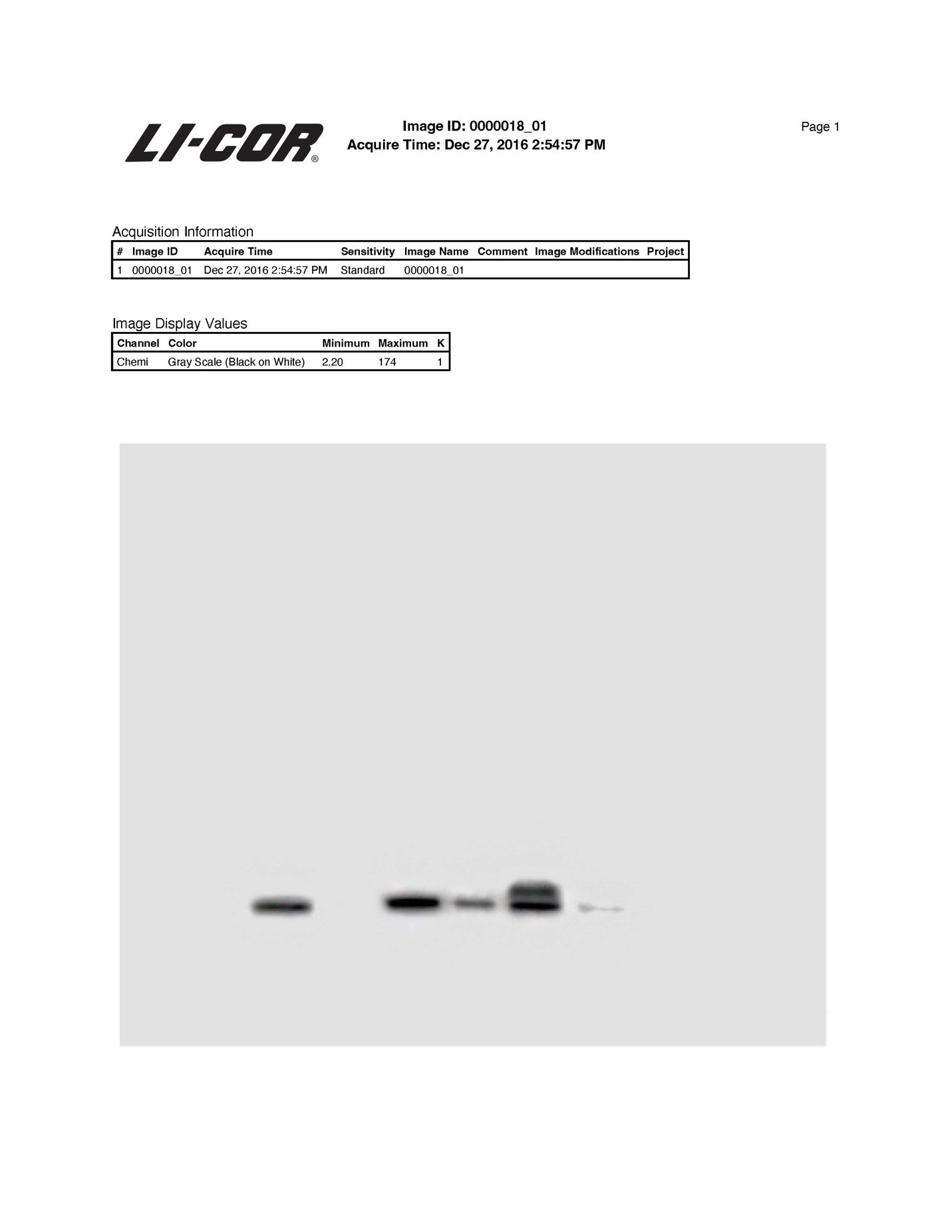


P21 western blot data (Uncropped image).


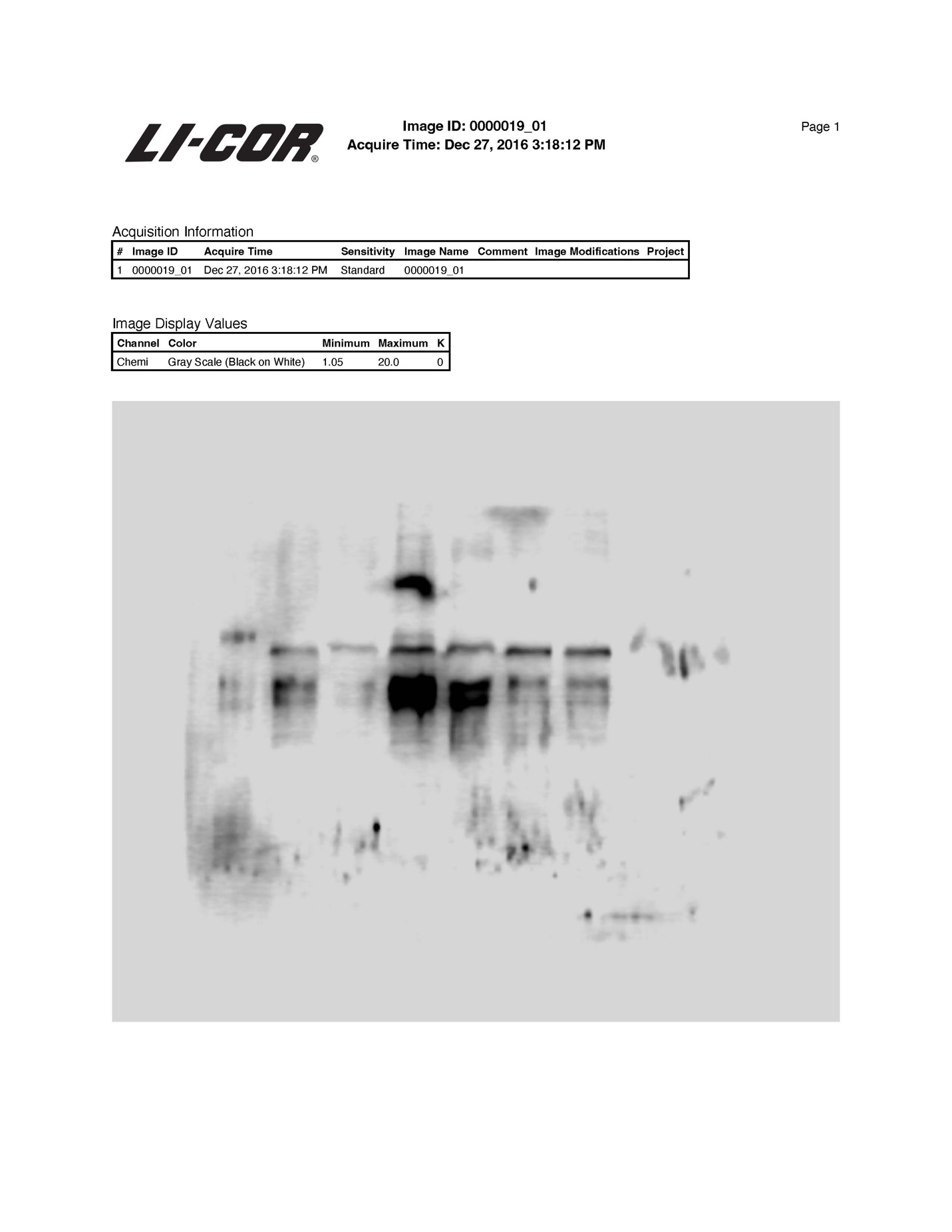


Cdc25C western blot data (Uncropped image).


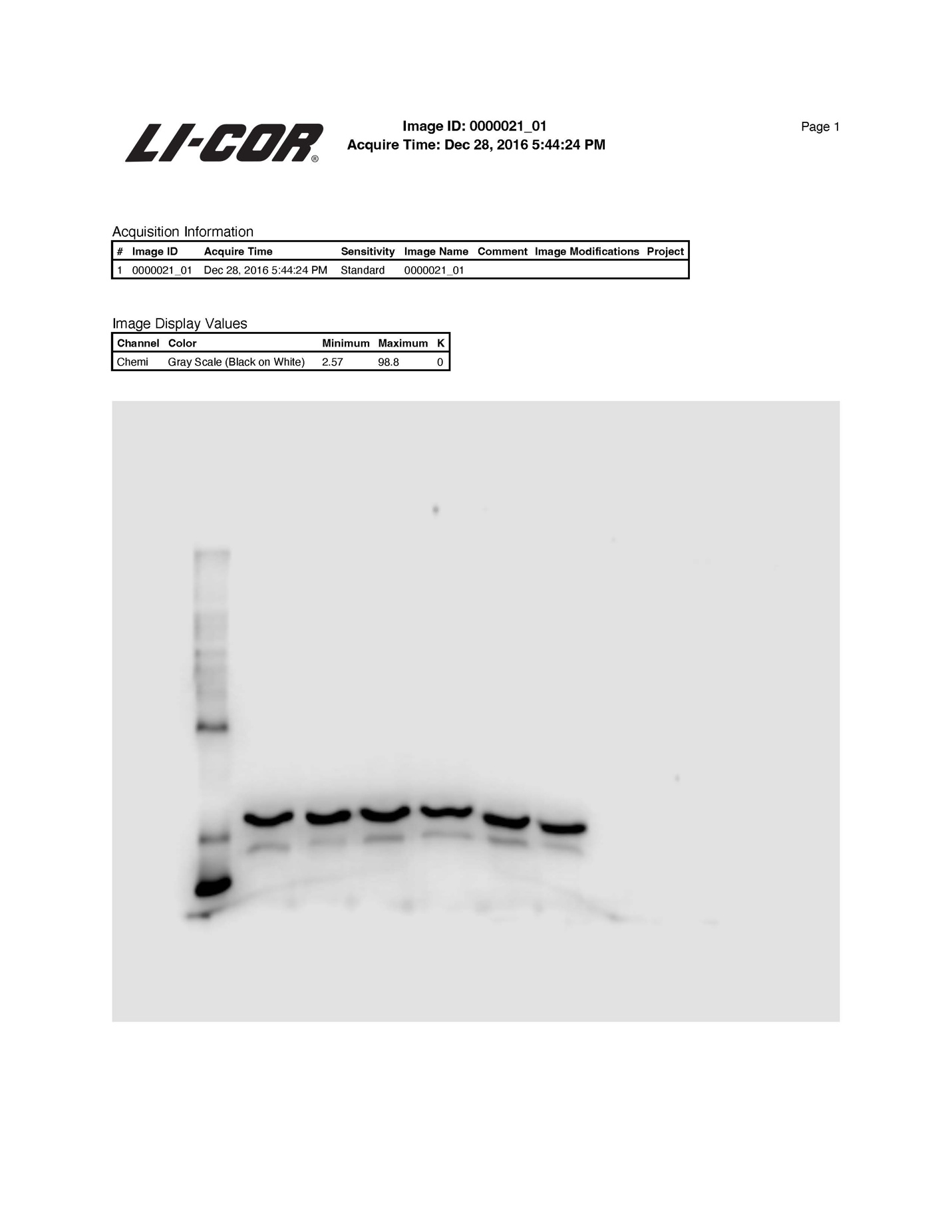


GAPDH western blot data (Uncropped image).
